# *Tanacetum* species: Bridging empirical knowledge, phytochemistry, nutritional value, health benefits and clinical evidence

**DOI:** 10.3389/fphar.2023.1169629

**Published:** 2023-04-20

**Authors:** Sohaib Khatib, Mansour Sobeh, Cecilia Faraloni, Latifa Bouissane

**Affiliations:** ^1^ Molecular Chemistry, Materials and Catalysis Laboratory, Faculty of Sciences and Technologies, Sultan Moulay Slimane University, Beni-Mellal, Morocco; ^2^ AgroBioSciences, Mohammed VI Polytechnic University (UM6P), Ben Guerir, Morocco; ^3^ Institute of BioEconomy, IBE, National Research Council, Florence, Italy

**Keywords:** Tanacetum, ethnopharmacology, ceramides, sesquiterpene lactones, pharmacology, toxicity, clinical evidence

## Abstract

**Introduction:** The *Tanacetum* genus consists of 160 accepted flowering species thriving throughout temperate regions, mainly in the Mediterranean Basin, Northern America, and southwestern and eastern Asia. *Tanacetum* species bear a long-standing record of use in the folk medicine of indigenous tribes and communities worldwide, along with multitudinous applications in traditional cuisines, cosmeceuticals, and agricultural fields.

**Methods:** Up-to-date data related to traditional uses, phytochemistry, biological activities, toxicity and clinical trials of the genus *Tanacetum* were systematically reviewed from several online scientific engines, including PubMed, Web of Science, Scopus, SciFinder, Wiley Online, Science Direct, and Cochrane library.

**Results and discussion:** Over the past three decades, 241 metabolites have been isolated from nearly twenty species, including phenolic acids, flavonoids, coumarins, fatty acids and alkanes, aldehydes, volatile compounds, and naphthoquinones. Some unique metabolites have also been identified, such as the ceramides tanacetamide (A-D) from *T. artemisioides*, pyrethrins from *T. cinerariifolium*, and sesquiterpene lactones from several species. However, these secondary metabolites are still poorly studied despite *in vitro* clues highlighting their colossal pharmacological properties, especially as hypotensive, neuroprotective, anticancer, and antimicrobial agents. Scientific studies have validated some traditional claims of the plant, such as antidiabetic, anticancer, anthelmintic, insecticide, antioxidant, and hepatoprotective activities, as well as against festering wounds, skin ulcers, urinary tract infections, and sexually transmitted diseases. Other ethnomedicinal uses for arthritis, gout, rheumatism, anemia, and as a litholytic, antivenom and diaphoretic have not yet been supported and would constitute the subject of further research.

## Graphical Abstract

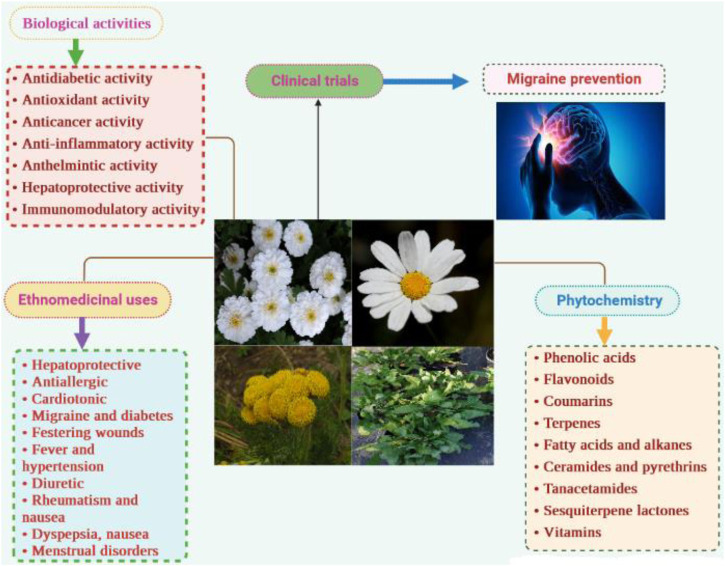

## 1 Introduction

Since the dawn of time, our ancestors have relied heavily on nature to meet their daily basic needs, such as shelter, foodstuffs, clothing, and medicines. Consequently, rich indigenous pharmacopeias have evolved through hit-and-miss, handed down, and maintained among healers and members of ethnic tribes and communities across generations. Today, it is estimated that more than 50% of modern therapeutic drugs are derived synthetically from herbal preparations and formulas, making them attractive templates for new drug leads (Heinrich, 2000; Drissi et al., 2022).

Species of the genus *Tanacetum* from the Asteraceae family bears a long history of traditional uses in various fields, including medicine, cosmetics, agriculture, and cuisines. They have been used ethnopharmacologically to treat many health-related conditions such as diabetes, migraine, cholecystitis, dyspepsia, nausea, diarrhea, hypertension, stomach pain and bloating, ringworms, and sexually transmitted diseases, among others (Molares and Ladio, 2009; Bouhlal et al., 2017; Ullah et al., 2019; Khatib et al., 2021).

A few taxa, mainly *T. balsamita* (Costmary), are still appreciated in the traditional cuisine of several countries, especially Italy, owing to their spicy odor and minty balsam aroma (Ghirardini et al., 2007; Cornara et al., 2014). For instance, leaves from costmary are used to prepare herbal tea, aromatize salads, omelets, soups, meats, and vegetable pies, and cosmetically to soothe and perfume the skin (Guarrera et al., 2005; Ghirardini et al., 2007). In the agricultural field, pyrethrum, from the dried and blended flowers of *T. cinerariifolium*, has long been used to repulse flying insects and ward off fleas and body lice, even before the chemistry of active metabolites emerged (Jeran et al., 2021).

Recently, phytochemical investigations have identified more than 240 secondary metabolites from the genus *Tanacetum*, including volatile compounds, phenolic acids, flavonoids, fatty acids and alkanes, aldehydes, and coumarins (Bagci et al., 2008; Benedec et al., 2016; Rezaei et al., 2017; Savci et al., 2020). Some dietary components such as carbohydrates and vitamins have also been found in the leaves, roots, and whole plants of *T. vulgare* and *T. densum* (Polle et al., 2001; Emre, 2021). Moreover, various unique compounds have exclusively been alarmed in the genus *Tanacetum*, such as the ceramides tanacetamide (A-D) **(72-74)** from *T. artemisioides*, pyrethrins **(113-118)** from *T. cinerariifolium*, and some sesquiterpene lactones **(119-139)** (Gonzalez et al., 1990; Hussain et al., 2005; 2005; Jeran et al., 2021). Thus, these metabolites could serve as crucial chemotaxonomic markers of the genus *Tanacetum*.

On the other hand, crude extracts and isolated metabolites have demonstrated various biological activities such as antidiabetic (Khan et al., 2018), antimicrobial (Kameri et al., 2019), cytotoxic (Coté et al., 2017), anthelmintic (Godinho et al., 2014), antioxidant (Bączek et al., 2017), and immunomodulatory activities (Jannesar et al., 2014), which are attributed to the produced synergetic effect or/and action of a single metabolite.

To our knowledge, this is the first comprehensive review of the genus *Tanacetum* since 2002 (Gören et al., 2002). Our review collates the fragmented ethnobotanical information on the genus during the last two decades to identify the validated medical applications and unveil the knowledge gaps to be fulfilled by further studies. We have also reviewed and updated the botanical features, phytochemical composition, pharmacological studies, toxicity, and clinical trials. A general discussion was established to link folkloric uses and secondary or/and primary metabolites potentially involved in the claimed uses, while shedding light on their unexplored therapeutic attributes.

## 2 Methodology

**FIGURE 1:**
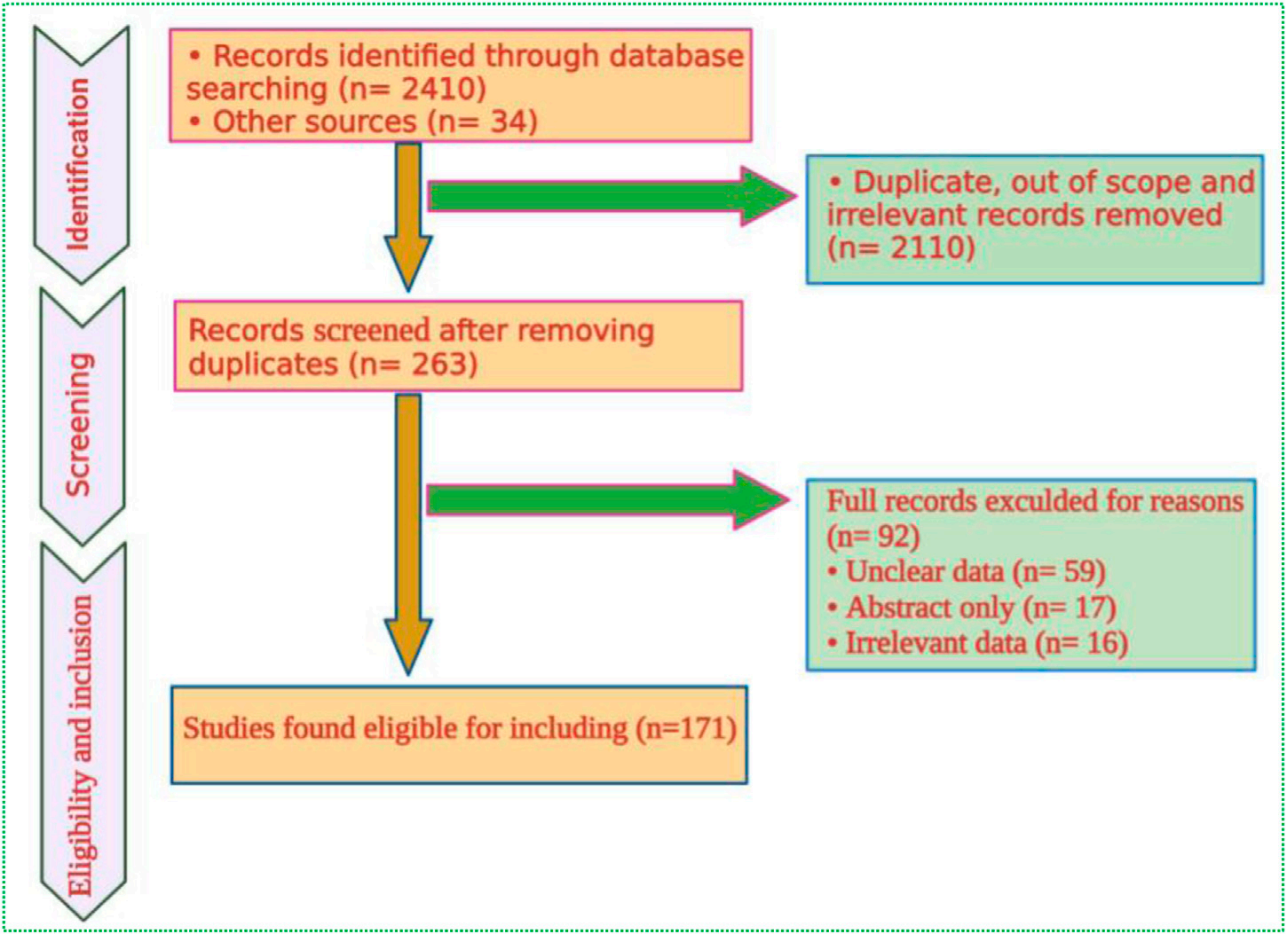
Flowchart of data screening, exclusion, and inclusion process.

Data were retrieved and systematically reviewed from several online scientific engines, including PubMed, Web of Science, Scopus, SciFinder, Wiley Online, and Science Direct (Figure 1). We have also reviewed the Cochrane Central Register of Controlled Trials to acquire the available evidence regarding randomized controlled trials (www.cochrane library.com). The key search words such as *Tanacetum*, ethnobotany, ethnoveterinary, geographical distribution, morphological features, phytochemistry, and biological activities, were used during the data search. The botanical names of *Tanacetum* taxa were validated using the World Flora Online (WFO, www.worldfloraonline.org) database.

## 3 Taxonomy, geographical distribution and IUCN status of *Tanacetum* spp

**FIGURE 2:**
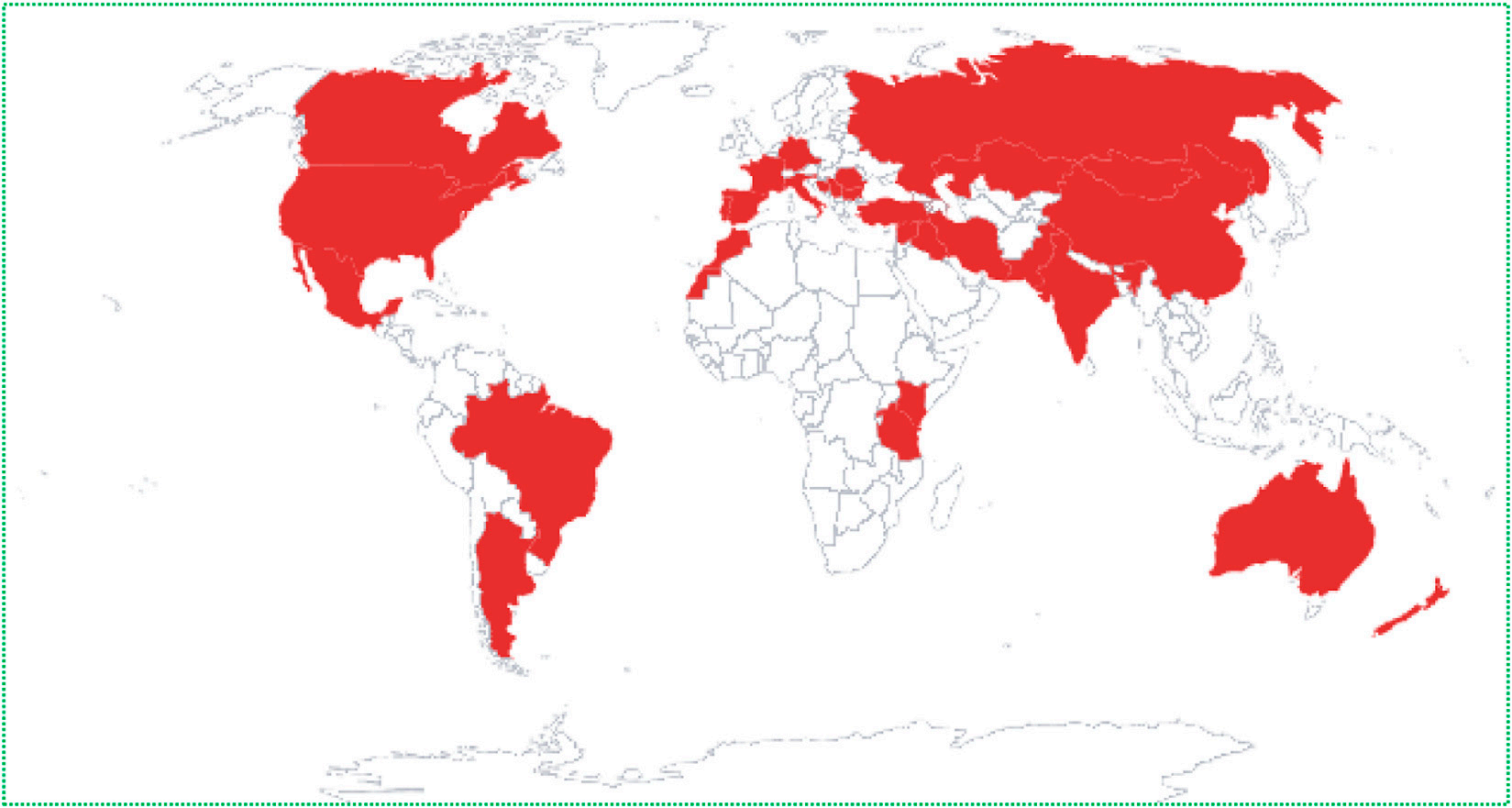
Geographical distribution of the genus *Tanacetum* 2023^**©**^.

The genus *Tanacetum* L. from the Asteraceae family is the third largest genus of the chamomile tribe Compositae–Anthemideae, consisting of about 160 species of flowering plants, after the two rich-species genera *Artemisia* L. (522 species) and *Anthemis* L. (177 species) (Sonboli et al., 2012; Moradi Behjou et al., 2022). According to The Plant List, 553 names have been granted to *Tanacetum* spp., including 179 accepted names, 206 synonyms, and 168 unresolved names (The Plant List, accessed on: 13 September 2022), while the worldfloraonline database has included 189 subordinate taxa (http://www.worldfloraonline.org/taxon/wfo-4000037526, accessed on: 13 September 2022) (Table 1). The species of the genus *Tanacetum* finds habitat throughout temperate regions, especially in the Mediterranean Basin region, some parts of northern America, and Southwestern and Eastern Asia, including Azerbaijan, Armenia, Iran, and Türkiye (Figure 2) (Moradi Behjou et al., 2022).

**TABLE 1:** Synonyms and geographical distribution of *Tanacetum* species used in the traditional medicine.

Tanacetum species	Synonyms	Distribution	References
*Tanacetum balsamita* L	*Balsamita major* Desf**	*T. balsamita* is native to Asia, Asia Minor, and Australia. Still, it has become naturalized in many regions of Europe, where it is well known and utilized in traditional medicine, especially in the Mediterranean basin’s countries such as Spain, Italy, Germany, England, Türkiye, and Romania	Cumo (2013), Faraloni et al. (2020), Bonetti et al. (2021)
	*Balsamita suaveolens* Pers**		
	*Balsamita vulgaris* Willd**		
	*Chamaemelum balsamita* (L.)E.H.L.Krause**		
	*Chrysanthemum balsamita* (L.) Baill**		
	*Chrysanthemum grande* (L.) Hook.f**		
	*Chrysanthemum grandiflorum* (Desf.) Dum.Cours**		
	*Chrysanthemum majus* (Desf.) Asch**		
	*Chrysanthemum tanacetifolium* (Desr.) Dum.Cours**		
	*Chrysanthemum tanacetum* Vis**		
	*Leucanthemum balsamita* (L.) Over**		
	*Matricaria balsamita* (L.) Desr**		
	*Pyrethrum majus* (Desf.) Tzvelev**		
	*Balsamita major* var. major*		
	*Balsamita major* subsp. *majo*r*		
	*Balsamita major* var. *tanacetoides* (Boiss.) Moldenke*		
	*Chrysanthemum balsamita* var. *tanacetoides* Boiss.*		
	*Tanacetum balsamita* subsp. balsamita*		
	*Tanacetum balsamita* var. balsamita*		
	*Tanacetum balsamita* subsp. *balsamitoides* (Sch.Bip.) Grierson*		
*Tanacetum vulgare* L	*Chamaemelum tanacetum* (Vis.) E.H.L.Krause**	This perennial herb is indigenous to temperate Europe and Asia, where it grows along roadsides, hedgerows, and trash areas. It was brought to North America for horticultural and medicinal uses but has since become wild in many American states	Juan-Badaturuge et al. (2009), Coté et al. (2017)
	*Chrysanthemum asiaticum* Vorosch**		
	*Pyrethrum vulgare* (L.) Boiss**		
	*Tanacetum boreale* Fisch. ex DC**		
	*Tanacetum crispum* Steud**		
	*Chrysanthemum vulgare* var. *boreale* (Fisch. ex DC.) Makino ex Makino & Nemoto*		
	*Dendranthema lavandulifolium* var. *tomentellum* (Hand.-Mazz.) Y.Ling & C.Shih*		
	*Tanacetum vulgare* subsp*. boreale* (Fisch. ex DC.) A*		
	*Tanacetum vulgare* var. *crispum* DC.*		
	*Tanacetum vulgare* subsp. vulgare*		
	*Tanacetum vulgare* f. vulgare*		
*Tanacetum artemisioides* Sch.Bip. ex Hook.f	*Chrysanthemum karakoramense* Kitam**	The species’ natural range extends from the Himalayas to western Tibet. It is a subshrub that primarily thrives in subarctic or subalpine biomes	Hussain et al. (2010)
*Tanacetum polycephalum* Sch.Bip	*Pyrethrum polycephalum* (Sch.Bip.) Sch.Bip. ex Boiss**	This herb grows mainly in Europe, Türkiye, Iraq, Iran, Caucasia, Turkmenistan, Afghanistan, Tibet, and Mongolia	Mahdavi et al. (2013)
*Tanacetum dolichophyllum* (Kitam.) Kitam	*Chrysanthemum dolichophyllum* Kitam**	This herb grows in the Uttarakhand Himalayas region at high altitudes	Haider et al. (2017)
*Tanacetum falconeri* Hook.f	No synonyms are recorded for this species	This species’ native habitat extends from northern Pakistan to the western Himalayas and western Tibet. It is primarily found in the subalpine or subarctic environment	Ghafoor (2002)
*Tanacetum fruticulosum* Ledeb	*Ajania fruticulosa* (Ledeb.) Poljakov**	The species occurs widely in the plains and hills of many parts of Central Asia and the Middle East of Hamedan, Iran, at a relatively high altitude (2000 m)	Weyerstahl et al. (1999)
*Tanacetum germanicopolitanum* (Bornm. & Heimerl) Grierson	*Chrysanthemum germanicopolitanum* Bornm**	This plant is native to Turkey (northern Anatolia) and thrives mainly in the chalky steppe	(web.archive.org; accessed on: 29 September 2022)
*Tanacetum cadmeum* (Boiss.) Heywood	*Achillea peroninii* Boiss**	This plant is represented by two subspecies, namely, *T. cadmium* subsp. orientale and *T. cadmeum* (Boiss.) Heywood subsp. *cadmeum*, both of which are endemic to Türkiye	Özek et al. (2007)
	*Achillea speciosa* Hayek**		
	*Chrysanthemum cadmeum* (Boiss.) Bornm**		
	*Chrysanthemum schwarzianum* Bornm**		
	*Pyrethrum cadmeum* Boiss**		
*Tanacetum zahlbruckneri* (Nab.) Grierson	*Chrysanthemum zahlbruckneri* Nábělek**	This species thrives in the Irano-Turanian region, including Türkiye, Iran, and Azerbaijan	Eyol et al. (2017)
*Tanacetum cinerariifolium* (Trevir.) Sch.Bip	*Chrysanthemum cinerariifolium* (Trevir.) Vis**	*T. cinerariifolium* is indigenous to the East Coast of the Adriatic Sea. It may also occur in the hilly areas of Croatia, Bosnia and Herzegovina, Montenegro, and northern Albania	Grdiša et al. (2009)
	*Chrysanthemum rigidum* Vis**		
	*Chrysanthemum turreanum* Vis**		
	*Pyrethrum cinerariifolium* Trevir**		
*Tanacetum cilicicum* (Boiss.) Grierson	*Chrysanthemum cilicicum* (Boiss.) Bornm**	The species is endemic to Türkiye	Şen et al. (2019)
	*Pyrethrum cilicium* Boiss**		
*Tanacetum parthenium* Sch.Bip	*Chamaemelum parthenium* (L.) E.H.L.Krause*	The species is endemic to the Balkan Peninsula but has also been introduced to Australia, Europe, Japan, China, and North Africa	Pareek et al. (2011)
	*Chrysanthemum parthenium* (L.) Bernh. *		
	*Chrysanthemum praealtum* Vent. *		
	*Dendranthema parthenium* (L.) Des Moul.*		
	*Leucanthemum odoratum* Dulac*		
	*Leucanthemum parthenium* (L.) Gren. & Godr.*		
	*Matricaria parthenium* L.*		
	*Parthenium matricaria* Gueldenst.*		
	*Parthenium matricaria* gesn. ex Rupr.*		
	*Pontia matricaria* Bubani*		
	*Pyrethrum buschianum* Sosn. *		
	*Pyrethrum demetrii* Manden.*		
	*Pyrethrum divaricatum* (Sosn.) Sosn.*		
	*Pyrethrum glanduliferum* Sommier & Levier*		
	*Pyrethrum grossheimii* Sosn.*		
	*Pyrethrum matricaria* gesn. ex Rupr.*		
	*Pyrethrum parthenium* (L.) Sm.*		
	*Pyrethrum sevanense* Sosn. ex Grossh.*		
	*Pyrethrum parthenium* (L.) J. E. Smith*		
*Tanacetum argyrophyllum* (K.Koch) Tzvelev	*Tanacetum argyrophyllum* var. argyrophyllum*	The species occurs widely in north-east Anatolia, Türkiye	Inceer et al. (2012)
*Tanacetum aureum* (Lam.) Greuter & al	Tanacetum chiliophyllum Sch.Bip**	The species and its varieties are endemic to South and West Asia, including Azerbaijan, Armenia, Iran, and Türkiye	Polatoğlu et al. (2012)
	*Tanacetum chiliophyllum* var. *monocephalum* Grierson**		
	*Pyrethrum chiliophyllum* Fisch. & C.A.Mey. ex DC**		
	*Matricaria absinthioides* Desr**		
	*Gymnocline chiliophylla* K.Koch**		
	*Chrysanthemum saxatile* B.Fedtsch**		
	*Achillea aurea* Lam**		
	*Tanacetum kochii* Sch.Bip**		
	*Tanacetum longipedunculatum* (Sost.)Tzuelev**		
	*Tanacetum tauricum* Sch.Bip**		
*Tanacetum coccineum* (Willd.) Grierson	*Chrysanthemum roseum* Adams*	The species is endemic to the Caucasus region between the Caspian Sea and the Black Sea	Zeng et al. (2021)
	*Pyrethrum roseum* var. *adamii* Trautv.*		
	*Pyrethrum roseum* var. roseum*		
*Tanacetum sinaicum (Fresen.)* Delile ex K.Bremer & Humphries	*Chrysanthemum sinaicum* (Delile ex DC.) Nábělek**	The species is endemic to the Middle East	Hegazy et al. (2015)
	*Pyrethrum santolinoides* DC**		
	*Santolina sinaica* Fresen**		
	*Tanacetum santolinoides* (DC.) Feinbrun & Fertig**		

**(Medium Confidence level); * (Low Confidence level).

It is worth noting that the genus exhibited considerable morphological variations encompassing perennial herbs and subshrubs, with the capitula either solitary or clustered in lax to dense corymbs and are either radiate or disciform-to-discoid (Sonboli et al., 2012). Due to its highly complex taxonomical history, phylogenetic position, and morphological intraspecific diversity, the infrageneric classification of the genus *Tanacetum* remains controversial within this medium-sized tribe (Sonboli et al., 2012; Moradi Behjou et al., 2022). For instance, Bremer and Humphries (1993) proposed a subtribal classification for the tribe based mostly on morphological traits, which molecular-phylogenetic studies later discovered to be substantially polyphyletic (Bremer, 1993; Oberprieler et al., 2007). Subsequently, molecular-phylogenetic investigations have excluded some species from the genus *Tanacetum* and were transferred to other circum-Mediterranean *Anthemidinae* genera, such as *Nananthea*, *Anthemis*, *Cota*, and *Tripleurospermum* (Sonboli et al., 2012).

According to the IUCN database, three *Tanacetum* taxa met the B2ab and C2a criteria of endangered species, and have recently been deemed as critically threatened, rendering their preservation and sustainability utterly necessary (www.iucnredlist.org; accessed on: 28 September 2022). These species are *T. ptarmiciflorum* Sch.Bip., *T. oxystegium* (Sosn.) Grierson, and *T. oshanahanii* “Marrero Rodr., Febles & C.Suárez” (www.iucnredlist.org; accessed on: 28 September 2022).

## 4 Morphological features of *Tanacetum* spp

**FIGURE 3:**
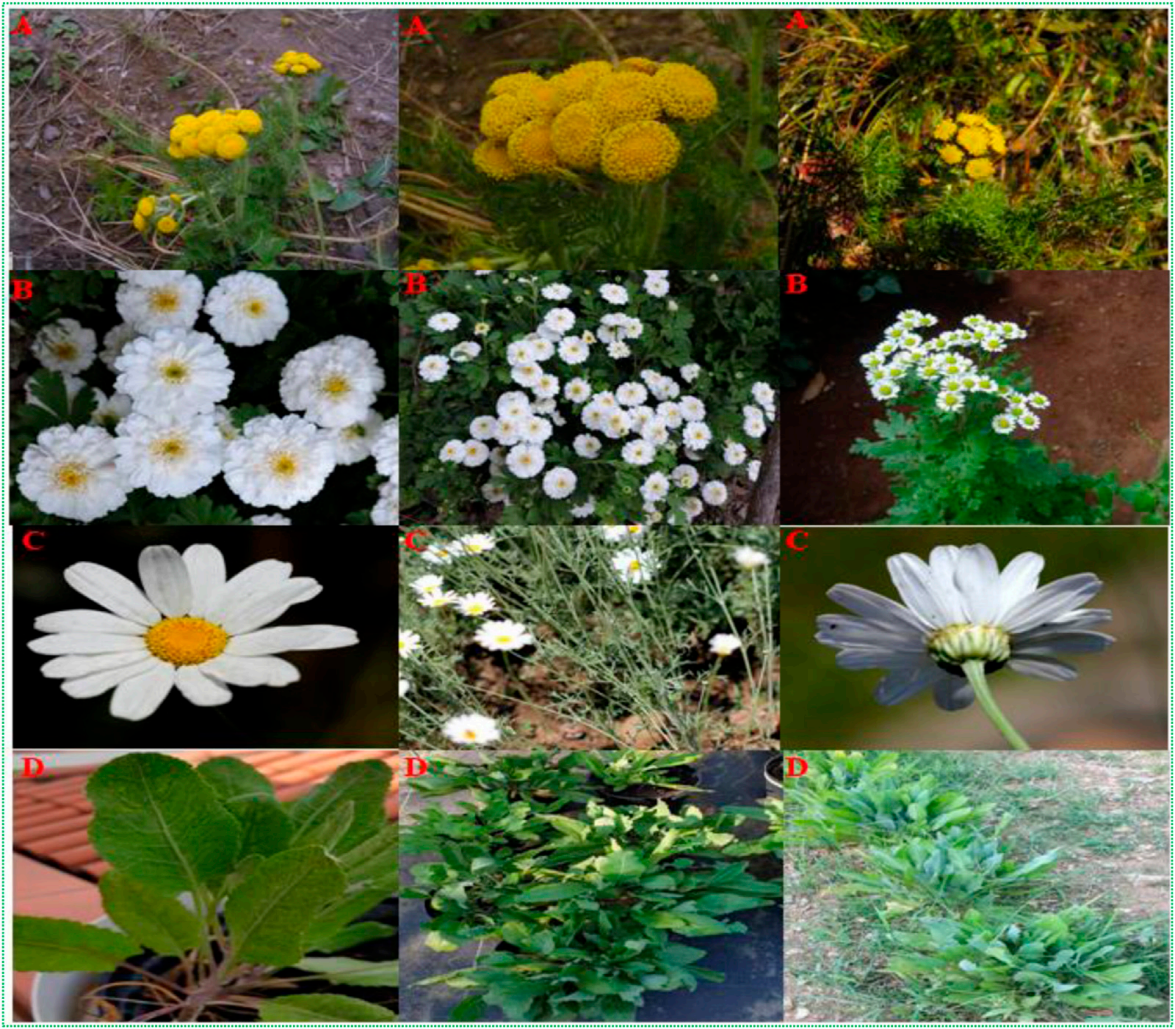
A collection of pictures of *Tanacetum spp*. **(A)** *T. dolichophyllum* (Kitam.) Kitam (https://sites.google.com/site/efloraofindia). **(B)** *T. parthenium* (L.) Sch.Bip (https://sites.google.com/site/efloraofindia). **(C)** *T. cinerariifolium* (Trevir.) Sch.Bip (https://sites.google.com/site/efloraofindia). **(D)** *T. balsamita* L., Florence, Italy, 2023^©^.

In botanical Latin, the generic name *Tanacetum* came seemingly from the Latin name *Athanasia* referring to “eternal life and immortality” since tansies were once sown between the grave clothes of the deceased to ward off vermin. Tansies are mostly perennial herbs, but a few can be annuals, evergreen, herbaceous perennials, or sub-shrubs. *Tanacetum* species vary in height from a few centimeters (5 cm) to 150 cm, with strongly scented, hairy, and occasionally silvery foliage. The leaves are alternate, basal and cauline, petiolate, or sessile, with the blades mostly obovate to spatulate (Figure 3). The flowers have distinct layers of phyllaries encircling their base and range in shape from flat to hemispherical. The fruit is a cypsela with ribs and glands that typically has a pappus at the end (http://www.efloras.org; accessed on: 28 September 2022). Tansies thrive naturally in well-drained sandy or coarse soils, requiring a limited amount of soil nutrients and humidity, and can be propagated by rooting stem cuttings under mist, tissue culture, vegetative splits, and seed propagation (Keskitalo, 1999).

## 5 Traditional and edible uses

**FIGURE 4:**
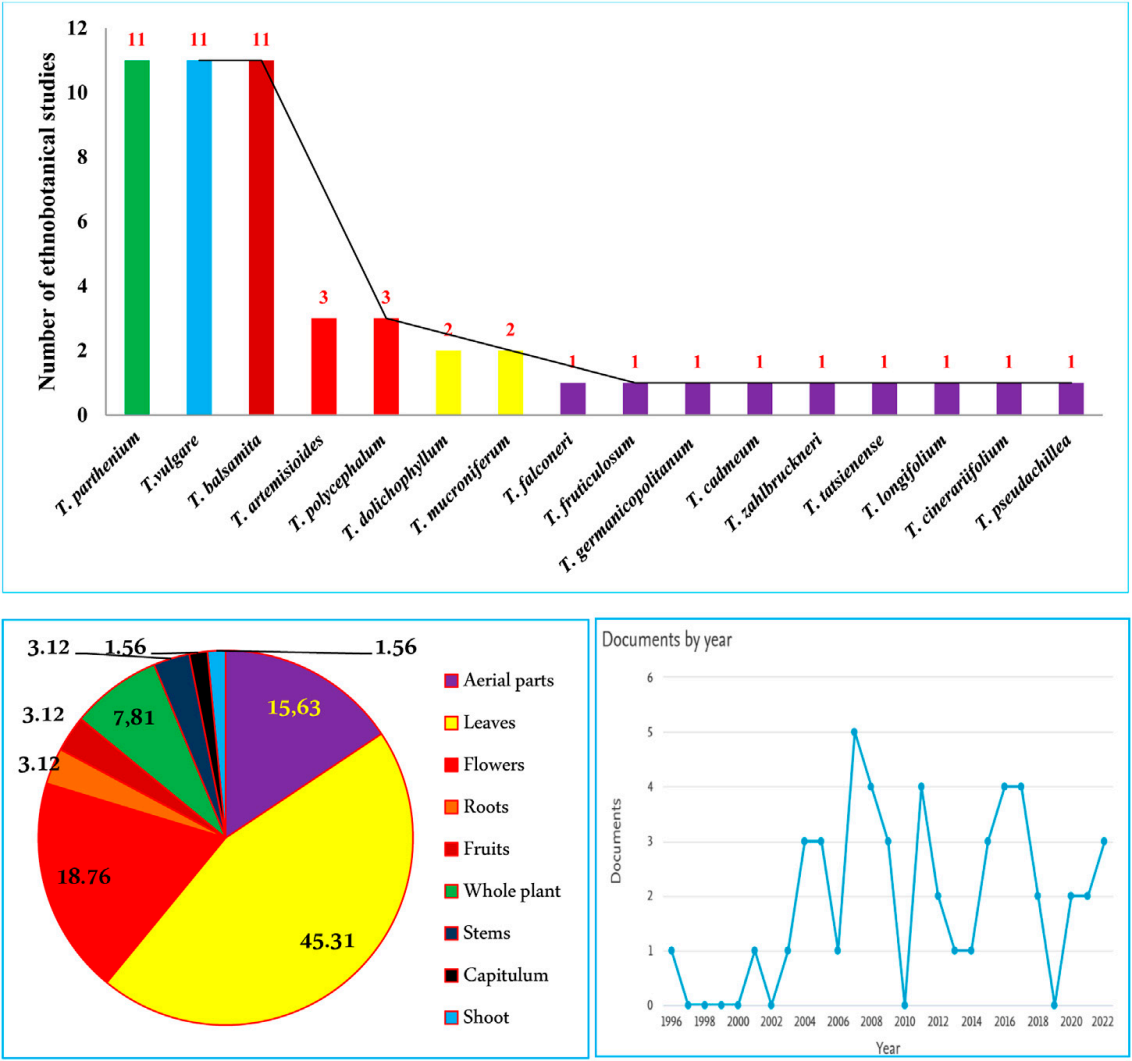
Number of ethnobotanical studies per *Tanacetum* species, main used parts, and publication trends 2023^**©**^.

Out of 160 *Tanacetum* species, ethnobotanical data for only sixteen taxa **(10%)** are available, while the remaining species have not yet been surveyed. Analysis of more than 50 ethnobotanical studies, undertaken worldwide, revealed that *T. vulgare*, *T. balsamita*, and *T. parthenium* are the major *Tanacetum* taxa used in ethnomedicinal practices. Meanwhile, the leaves (**45.31%**), flowers (**18.76%**), and aerial parts (**15.63%**) are the predominant parts (Figure 4). In Ayurvedic medicine, mountainous communities drank the juice made from crushed and boiled roots of *Pleurospermum* and *Tanacetum* spp. three to four times daily to cure gastritis and stomachache. The underground parts are also cleansed, cut into small pieces, and chewed for arthritis and fever (Abbasi and Bussmann, 2021). The following subsections and Table 2 compiled exhaustive details about the traditional/ethnopharmacological uses of the sixteen taxa, including their vernacular names, used parts, ethno-preparations, and routes of administration.

**TABLE 2:** Comprehensive overview of the ethnopharmacological uses of *Tanacetum* spp.

Vernacular names	Used part	Ethno-preparations	Ethnobotanical uses	Administration route	Country	References
***T. artemisioides* Sch.Bip. ex Hook.f**						
*Zawil*	AP	Infusion	Diabetes	Internal	Pakistan	Ullah et al. (2019)
*Zawil*	Fl	Powder blended with oil and sugar	The flowers are powdered, mixed with oil and sugar, and swallowed to cure the flu	Internal	Pakistan	Ali et al. (2019)
*Zawil*	Le, Fr	Powder	The powder prepared from leaves, branches, and fruit is used to treat hepatitis and relieve chest pain	Internal/External	Pakistan	Ibrar and Hussain (2010), Singhal et al. (2016)
***T. argyrophyllum* var. *argyrophyllum***						
Nr	Nr	Nr	Migraine, neuralgia, anorexia, and rheumatism, and as a vermifuge	Internal/External	Türkiye	Akpulat et al. (2005)
*Nalbant*	R	Burnt and mixed with sulfur, gunpowder, and butter	Wound healing, scabies	External	Türkiye	Sezik et al. (1997)
*Yavşan*						
***T. balsamita* L**						
*Boldo*Menta romana	Nr	Nr	Hepatic and stomach problems	Internal	Argentina	Molares and Ladio (2009)
*Kalofer*	Le	A vinegar-based mixture with the plant leaf	General strengthening	Nr	Bulgaria	Nedelcheva (2012)
*Shahsparam*	Le	Nr	Stomach pain and bloating	Internal	Iran	Ebadi and Eftekharian (2019)
*Erba di San Pietro*	Le	Cooking	Aromatize salads, omelets, and liqueurs, particularly on Easter Day. They have also been cooked with cheese, eggs, cloves, garlic, and mallow leaves	Internal	Italy (Central Italian)	Ghirardini et al. (2007)
*Erba amara*						
*Erbadella Madonna*						
*Erba amara balsamica*	Le	Infusion, tisane	Bile acid deficiency, cholecystitis, dyspepsia, sedative, antispasmodic for insomnia and cough, carminative, and as a diuretic	External	Italy (Southern Italy)	Guarino (2008)
*Erba di Santa Maria*						
*Erba di San Pietro*						
*Erba menta*						
*Erba della Madonna “lilla”*	Le	Dried leaves added in the bath	To strengthen the skin of newborns, also as a skin toner and performing (ritual)	External	Italy (Central-Eastern)	Pieroni et al. (2004)
*Nr*	AP	Chewing	Oral hygiene, decorative purposes	External/Internal	Lithuania	Pranskuniene et al. (2019)
*Moteržole*	Le	Infusion, cooked	Nausea, diarrhoea, and women’s disorders	Internal	Lithuania	Karpavičienė (2022)
*Balsam*	Nr	Herbal tea	Lower high blood pressure, as a hypoglycemic drink, and to reduce cough symptoms	Internal	Morocco	Bouhlal et al. (2017)
*Nr*	Nr	Nr	Anthelmintic	Internal	Spain	Agelet et al. (2000)
*Kaloper*	Le	Tea	Female problems during the menopause, and intense migraines	Internal	Serbia	Jarić et al. (2015)
		Spice	Carminative, and as a component of individual’s diet			
***T. cadmeum* (Boiss.) Heywood**						
Nr	AP, Fr	Infusion, powder, boiled	Cold, chest pain, carminative, ulcer, and stomachache	Internal/External	Türkiye	Türker (2018)
***Tanacetum coccineum* (Willd.) Grierson**						
*Sendel*	L, Fl, St	Decoction	Sterility	Internal	Türkiye	Altundag and Ozturk (2011)
***T. cinerariifolium* Sch.Bip**						
*Pareto*	WP	Nr	The plant is used as pesticidal to manage pests and as a veterinary remedy against ticks	External	Tanzania	Qwarse et al. (2018)
***Tanacetum corymbosum* (L.) Sch.Bip**						
Nr	WP	Nr	Digestive disorders, gastritis, and parasitic intestinal worms	Internal	Türkiye	Altundag and Ozturk (2011)
***T. dolichophyllum* (Kitam.) Kitam**						
*Amritdhara-ghas*	Le	Pills	The leaves are formed into a tablet the size of an almond nut and taken orally with water	Internal	India	Kumar et al. (2009)
*Khampaserpo Seigmanlo*	Fl^**α**^, Le^**β**^, R^**γ**^, WP^**δ**^	Nr	Intestinal worms^**α,β,γ**^, stomachache^**β**^, Indigestion^**β**^, and fever^**δ**^	Internal	India	Gairola et al. (2014)
*Lidd guggli*						
*Amritdhara-ghas*						
***T. emodi* R.Khan**						
*Phematso*	Sht	Nr	Antiseptic	Nr	India	Gairola et al. (2014)
***T. falconeri* Hook.f**						
*Zoon*	Le, Fl	Nr	Asthma and respiratory problems, flatulence, ring worms, and stomachache	Internal	Pakistan	Khan and Khatoon (2008)
***T. fruticulosum* Ledeb**						
*Dermene shah*	Le (Fresh)	Nr	Abdominal pain, stomachache, and flatulency	Internal	Iran	Safa et al. (2013)
***T. germanicopolitanum* (Bornm. & Heimerl) Grierson**						
Nr	Nr	Nr	Appetizing, tonic, and gynecological problems	Internal	Türkiye	Türker (2018)
***T. longifolium* Wall. ex DC.**						
*Ban chai*	R	Powder	The powdered roots suspended in milk are orally taken to alleviate stomach pain	Internal	India	Dutt et al. (2015)
***T. mucroniferum* Hub.-Mor. & Grierson**						
Nr	Fl	Nr	Anti-inflammatory, cold, flu, stomachache, edema, kidney problems, and insomnia	Internal	Türkiye	Türker (2018)
*Wurmkruid*	Nr	Nr	Poultice for scorpion bites, stomachache, typhoid fever, infantile spasms, and influenza	External/Internal	Nr	Van Wyk and Gorelik (2017)
*Wurmbos*						
*Miskruid*						
***T. nubigenum* Wall. ex DC.**						
Nr	WP	Decoction	Fever	Internal	India	Chanotiya et al. (2006)
***T. parthenium* (L.) Sch.Bip**						
*Manzanilla*	Le, Fl	Infusion	Vaginal discharge, stomachache	Internal	Argentina	Kujawska and Schmeda-Hirschmann (2022)
*Altamisa casera*						
*Feverfew*	AP	Paste	Feverish horses	Internal	Canada	Lans et al. (2006)
*Erba amara vera*	Fl, WP	Decoction	The whole plant is used to alleviate menstrual pain, while the flower decoction served to treat skin ulcers and contusions	External	Italy	Guarino (2008)
*Babune gavi*	Le	Decoction	Gastric ailments, sedative, fever, and nerve system relaxant	Internal	Iran	Rajaei and Mohamadi (2012)
*Mokhalaseh*	Le, St	Nr	Analgesic	Internal	Iran	Basati et al. (2019)
*Santamaría*	AP, Le	Infusion	Abortifacient	Internal	Mexico	Andrade-Cetto (2009)
*Babouneh gavi*	Nr	Nr	Ulcerative colitis	Internal	Iran	Karimi et al. (2017)
*Botonets*	FAP	Tisane	Antiseptic in cows after calving	Internal	Spain	Bonet and Vallès (2007)
*Papatya*	Le, Fl	Decoction	Bronchitis, common colds	Internal	Türkiye	Karaköse (2022)
*Papatya*	Fl	Tea	Stress-related disorders, sedative	Internal	Türkiye	Eruçar et al. (2022)
*Gümüşdüğme*						
*Moteržole*	Le	Cooked with omelets	Bowel diseases	Internal	Lithuania	Karpavičienė (2022)
***T. polycephalum* Sch. Bip**						
*Mokhalaseh*	Le, St	Nr	Sedative	Nr	Iran	Abbaszadeh et al. (2019)
*Samsa*	Le	Nr	Anti-inflammatory, anti-hemorrhoid and sting	External	Iran	Ghasemi Pirbalouti et al. (2012)
*Daramey-e Pus*	AP	Decoction	The aerial part decoction is blended with that of the *Thymus* and *Achillea* and used to treat gastroenteritis	Internal	Iran	Mosaddegh et al. (2012)
*Borzhan*	Ca	Decoction	Flu and cold	Internal	Iraq	Kawarty et al. (2020)
***T. pseudachillea* C.Winkl**						
*Dastarbosh*	AP	Decoction	Laxative	Internal	Uzbekistan	Khojimatov et al. (2020)
***T. sinaicum* (Fresen.) Delile ex K.Bremer & Humphries**						
Nr	Nr	Nr	Migraines, fevers, arthritis and bronchitis, and stomach ailments	Internal/Internal	Egypt	Hegazy et al. (2015)
***T. tatsienense* var*. tanacetopsis* (W. W. Smith) Grierson**						
Nr	WP	Nr	Rheumatism, dyspepsia (the upper abdomen pain), and blood bleeding	External/Internal	China	Cheng et al. (2022)
***T.vulgare* L**						
*Palma-crespa*	Le, Fl	Bottled	Rheumatism	External	Brazil	Tribess et al. (2015)
*Catinga-de-mulata*						
*Água da colônia*	WP	Tea	The plant’s tea is swallowed daily against dizziness and for its calming effects	Internal	Brazil	Chagas Nogueira et al. (2016)
*Catinga-de-mulata*						
*Catinga demulata*	Le		Headache	Nr	Brazil	Davis et al. (2021)
*Tanaceto*	Le, Fl, Sd	Nr	Anti-inflammatory and helminth infections	Internal	Brazil	Holetz et al. (2002)
*Erva dos vermes*						
*Tanaceto*	Le, Fl (Fresh)	Infusion	The infusion of flowers suspended in milk or water or intake of a spoonful of crushed flowers with honey is used as a vermifuge, while the leaves soaked in a liqueur called *arquébuse* served as digestive	Internal	Italy	Danna et al. (2022)
*Fiori della Madonna*						
*Archebùe*						
*Boutòn du vers*						
Nr	Le (Fresh)	Infusion	Fresh leaves soaked in alcohol were used for the digestive tract	Internal	Italy	Pieroni and Giusti (2009)
*Balssem*	Le	Infusion	Diabetes, anemia, and hypercholesterolemia	Internal	Morocco	Chaachouay et al. (2019b)
*Balssem*	Le	Infusion	Type 1 diabetes	Internal	Morocco	Chaachouay et al. (2019a)
*Vratič*	Le, Fl	Nr	Anthelmintic (Worms and tapeworms)	Internal	Türkiye	Jarić et al. (2015)
*Tanarides*	AP	Tisane	Aphrodisiac in sows	Internal	Spain	Bonet and Vallès (2007)
*Hasheshet eldood*	AP	Infusion	Digestive stimulants, coughs, respiratory problems, gastritis, neurological and venereal diseases, wounds, and as a repellent of ants	Internal/External	Syria	Khatib et al. (2021)
Nr	Fl	Nr	Food additives and preservatives	External	Russia	Shikov et al. (2017)
***T. zahlbruckneri* (Nábělek) Grierson**						
Nr	AP, Le	Decoction	Flu, cold, asthma, and styptic	Internal/External	Türkiye	Türker (2018)

Abbreviations: Le, Leaves; St, Stems; AP, aerial parts; WP, whole plant; Nr, Not reported; Fl, Flowers; FAP, flowered aerial parts; Ca, Capitulum; Fr, Fruits; R, roots; Sht, Shoot; Sd, Seeds.

### 5.1 *T. balsamita* (costmary)

In Southern Europe, the leaves decoction from costmary had been applied as an insect repellent for cattle and children and as an insecticidal agent. Costmary had also been used to disguise unpleasant odors in houses and to disseminate a pleasant smell in closets (Cumo, 2013). By 1614, the balsamic scent of costmary inspired *Fra’ Angiolo Marchissi* to create a fragrant preparation in water with *Ceylon cinnamon*, rosemary, and mint. This concoction was used for coughs, colds, and its relaxing properties. Therefore, this distilled preparation was commonly known as “Anti-hysteric Water” (Nelli and Ena, 2012). Costmary had once been employed as a beer flavoring, but in the 15th century with the extensive usage of hop (*Humulus lupulus*), it gradually fell into decline for this purpose (Nelli and Ena, 2012).

Recently, Sõukand and Pieroni, (2016) stated that the aboriginal inhabitants in the *Hutsuls* of Bukovina area used the alcohol infusion of *T. balsamita* (costmary) flower buds and leaves topically to treat heart diseases and festering wounds. In some cases, they utilized fresh aerial parts soaked in hot water to cure old and deep wounds and furuncles (Sõukand and Pieroni, 2016).

In the Persian pharmacopeia, the leaves and flowerheads in the form of decoction, infusion, and floral water of costmary have been used as a general tonic, antiallergic, anticancer, hepatoprotective, sedative, flatulent, and cardiotonic, whereas in Serbia, the leaves’ tea aids to ease terrible migraines and female issues during the menopause (Hassanpouraghdam, 2009; Jarić et al., 2015; Hassanpouraghdam et al., 2022). Moreover, the decoction of the leaves and stems was applied topically as a rheumatism ointment, antipyretic, and a menstrual regulator (Güneş and Özhatay, 2011).

In Northern Istria, the indigenous population breathed the ensuing vapors of *T. balsamita* scorched leaves with rose petals and wormwood on June 21st for their relaxing properties (Pieroni and Giusti, 2008). In Southern Italy, locals ingested an infusion from costmary leaves against bile insufficiency, cholecystitis, and nervous dyspepsia, and for its sedative, antispasmodic, anti-inflammatory, and anti-insomnia properties (Guarrera et al., 2005; Ghirardini et al., 2007; Guarino, 2008; Vitalini et al., 2015). In Turkish folk medicine, two teacups from an infusion of *T. balsamita* leaves are prescribed thrice daily for three consecutive weeks against diabetes (Dalar, 2018).

Intriguingly, costmary still finds application in the traditional cuisine of central Italy owing to its distinctive bitter taste; the minty-lemony leaves served to aromatize salads, omelets, vegetable pies, liqueurs, and as a component of the filling of Tortelli, especially on Easter Day (Ghirardini et al., 2007; Cornara et al., 2014). They have also been employed to flavor garlic cloves, mallow leaves, cheese, and eggs (Ghirardini et al., 2007).

The macerated water of *T. balsamita* and *Santolina etrusca* has been frequently used to make fragrant water on *St. John’s* evening to soften and perfume the skin (Guarrera et al., 2005). In accordance with tradition, the most appropriate day for harvesting this plant is June 24, *St. John’s Day,* and for this reason, this medicinal plant is also called *St. John’s* herb. Perhaps, the tradition follows the findings that this period typically coincide with the highest balsamic period of costmary, featured by an intense aromatic flavor (Pukalskas et al., 2010).

### 5.2 *T. vulgare* (tansy)

In Syria, *T. vulgare* is widely known as *“*Hasheshet eldood”, referring to its miraculous ability to eradicate internal worms. Thereby, it is used to remove parasitic worms and externally for its wound healing properties. Indigenous villagers also used to swallow an infusion from the aboveground parts to heal neurological and venereal conditions, coughs, gastritis, and respiratory tract infections. It is said to have repellent properties against some kinds of ants owing to its aromatic odors (Khatib et al., 2021).

In Russian folk medicine, tansy has a long-established use against diarrhea and intestinal worms (*Enterobius* and *Ascaris*), as well as an antipyretic and diaphoretic agent (Shikov et al., 2014). Interestingly, 10 g of the decocted flowers in 200 mL of water is believed to have anthelmintic and choleretic effects when consumed at a dose of 1 tablespoon daily (Shikov et al., 2014). Externally, the poultice from the whole plant is applied for sprains, swellings, contusions, gout, and some eruptive skin conditions (Abad et al., 1995; Shikov et al., 2014). Moreover, the leaves, flowers, and whole plant infusion are mentioned in preventing and treating rheumatism, anemia, hypercholesterolemia, kidney weakness, migraine, and hysteria (Shikov et al., 2014; Tribess et al., 2015; Chaachouay et al., 2019b).

In the Russian Pharmacopoeia, the dried flowers of tansy are used as a substitute for cinnamon and ginger. They can also be used to preserve meat and add flavor to fish, meat, and beverages. Leaf and flower parts are used as tea substitutes, and in beer as hop substitutes (Shikov et al., 2017).

### 5.3 *T. artemisioides*

The geographical restriction of *T. artemisioides* in Pakistan allowed the emergence and spread of rich beliefs and practical knowledge within the mountains tribes. In the Kurram Valley, the locals call this species *“Zawil”* and *“Zoon in Gilgit”*, and they are used to relieve the flu by mixing and consuming powdered flowers with oil and sugar (Hussain et al., 2010; Ali et al., 2019). They also used powdered leaves and fruits to alleviate and treat diabetes, high blood pressure, kidney, headache, fever, hepatitis, abdominal disorders, ringworm, and flatulence (Hussain et al., 2010; 2018; Ullah et al., 2019). Moreover, a glance at the existing data gathered from different geo-cultural areas indicated a typical usage of several *Tanacetum* species for diabetes management, including *T. artemisioides*. For instance, ethnic groups from *Khyber Pakhtunkhwa*, Pakistan, used to deal with diabetes by preparing and consuming 10 g water infusion of *T. artemisioides* aerial parts (Ullah et al., 2019).

### 5.4 *T. cinerariifolium* (dalmatian pyrethrum)

Since the 19th century, Dalmatian pyrethrum has been widely cultivated to repel and control mosquitoes and body lice on both animals and humans before the chemistry of active ingredients (Grdiša et al., 2009). The early 20th century marked the pioneering discovery of the active ingredients in Pyrethrum products by the German chemist Herman Staudinger and the Croatian scientist Lavoslav Ružička (Grdiša et al., 2009). Today, Kenya, Rwanda, and Tanzania are the leading producers of pyrethrum in the world, accounting for nearly 90% of the world’s output and 85% of exports; the ground pyrethrum flowers are manufactured and commercially sold as *“Dalmatian Insect Powder”* (Hitmi et al., 2000; Grdiša et al., 2009; Grdiša et al., 2022).

In the *Mbulu* district of Tanzania, agropastoralists call this species *“Pareto”*, and they grow it to control field pests and veterinary to manage ticks (Qwarse et al., 2018). It is also used by herbalists in central Morocco to control vector-borne diseases (EL-Akhal et al., 2021). Medically, ethnic communities in south India are still using the whole plant as an antidote for poisoning (Kumar et al., 2019). In North-Eastern Morocco, *T. cinerariifolium* is widely known as *عود العطاس*)) by local inhabitant, and they orally consume the stem infusion at a dose of one tablespoon daily to treat kidney stones (Bencheikh et al., 2021).

### 5.5 *T. parthenium* (feverfew)

In Southern Brazil, this species is known by rural communities as *“Rainha-das-ervas”* and *“Artemisia”*. The leaves and flowers decoction is used to jump-start and relieve menstrual pain, stomachache and infections (Tribess et al., 2015). In northwest Greece, this species is widely used against digestive system inflammations, puerperal fever, rheumatism, and arthritis, and as diaphoretic, emmenagogue, tonic, and stimulant (Vokou et al., 1993).

The local people in the Irano-Turanian region call this species *“Babune gavi”* and *“Colous”*, and they used the leaves decoction to cure fever and gastric disorders and as a sedative and nerve relaxant (Rajaei and Mohamadi, 2012). To ease toothache, they prepared a decoction of crushed roots and put it on the tooth (Delfan et al., 2014). Feverfew is called *“Santamaría"* in Mexican folk medicine; high doses from the aerial parts and leaves infusion are orally taken to induce abortion (Andrade-Cetto, 2009). The roots are mixed with honey and vinegar and used as a litholytic for bladder stones (Ahmed et al., 2016).

### 5.6 *T. polycephalum*

In Northern Iraq, this species is known as *“Borzhan”*. The locals consumed one glass of the flowers decoction on an empty stomach for cold and flu (Kawarty et al., 2020). The water decoction of the aerial parts is mixed with the *Thymus* and *Achillea* and used for gastroenteritis (Mosaddegh et al., 2012). It is also used as a traditional Iranian remedy for hemorrhoids and inflammation (Ghasemi Pirbalouti et al., 2012).

### 5.7 *T. nubigenum*

The available data regarding this species revealed that the local inhabitants in the Indian Himalayas privileged this species for preparing fragrant materials and incense owing to its distinguished pleasant smell (Beauchamp et al., 2001; Khan et al., 2018). They also used whole plant decoction to alleviate and treat fever (Chanotiya et al., 2006).

### 5.8 *T. macrophyllum*

The water infusion of *T. macrophyllum* flowers is reportedly used for earache (Kazancı et al., 2020).

### 5.9 *T. zahlbruckneri*

Indigenous villagers from the Eastern Anatolia region of Turkey drank the decoction of the aerial parts for flu and cold (Mükemre et al., 2015).

### 5.10 *T. cadmeum*

A single ethnobotanical study reported that some Turkish people chew the above-ground parts of this plant for stomach ulcers (Altınbaşak et al., 2018).

### 5.11 *T. ferulaceum*

The only traditional medicinal indication for this species is treating gastric ulcers (Kumar and Tyagi, 2013). Further ethnopharmacological studies are needed to document the traditional medicinal uses related to this species.

### 5.12 *T. corymbosum*

The whole plant is mainly used against digestive disorders, gastritis, and parasitic intestinal worms (Ciocarlan et al., 2021; Ivănescu et al., 2021).

### 5.13 *T. sinaicum* (pyrethrum santolinoides)

The species is native to the Middle East and has traditionally been used for migraine, fever, stomach disorders, arthritis, and bronchitis (Hegazy et al., 2015).

### 5.14 *T. argyrophyllum*

The species has been traditionally used to treat migraine, neuralgia, anorexia, and rheumatism and as an anthelmintic (Akpulat et al., 2005).

## 6 Ethnoveterinary applications

Ethnoveterinary medicine (EVM) refers to a complex multifaceted system of beliefs, skills, techniques, and practices used to prevent, treat, and promote the health of husbandry livestock and other income-generating animals (McGaw and Eloff, 2008; McGaw and Abdalla, 2020). Even though these practices have steadily been handed down across generations, a myriad of ethnoveterinary surveys stated that the know-how pertaining to livestock healthcare is mainly retained by elderly people (Bartha et al., 2015; Jamil Ahmed and Murtaza, 2015; Eiki et al., 2021; Güler et al., 2021; Khatib et al., 2022b). Thereby, this ancestral medical wealth may be doomed to disappear with the death of their practitioners.

As such, tremendous efforts are poured into preserving and documenting ethnospecies used in the ethnoveterinary practices of several countries to sustain their empirical medical knowledge for posterity. In the Kyrgyz Republic, nomadic herders are used to cure their livestock by preparing an infusion of *T. vulgare* flowers, which is subsequently orally or topically administered to cattle to treat parasites, scabies, and osteoporosis (Aldayarov et al., 2022). In Spain, people believed that the tisane made from aerial parts of *T. vulgare* has aphrodisiac effects in sows (Bonet and Vallès, 2007). In the rural areas of Serbia, *T. vulgare* aerial parts tea is allegedly prescribed as a remedy to cleanse animals with maggot-infested wounds (Jarić et al., 2014).

*T. parthenium* is frequently combined with other plants as part of herbal formulas and administered to cattle to cure a variety of conditions. For instance, in the traditional medicine system of Canada, equal amounts of the dried aerial parts of *T. parthenium* (Widely known as feverfew), *Filipendula ulmaria* (L.) Maxim., *Achillea millefolium* L., and *Salix alba* L. bark or leaves are blended to make a poultice fed to feverish horses (Lans et al., 2006). In Spain, the tisane from the flowering aerial parts of *T. parthenium* mixed with *Plantago lanceolata* L., *Lippia triphylla* (L'Hér.) Kuntze and *Triticum aestivum* L. is orally fed to cows as a *postpartum* antiseptic (Bonet and Vallès, 2007).

In summary, several *Tanacetum* taxa have proved their efficacy in preventing, treating, and promoting livestock health. As such, they may constitute a promising alternative for poorer livestock keepers due to their affordability, easy accessibility, and effectiveness. They may also unlock avenues for new antimicrobial agent discovery and remain a choice for rich livestock raisers, especially if the animal’s market value does not meet the cost of veterinary care.

## 7 Phytochemistry

**FIGURE 5:**
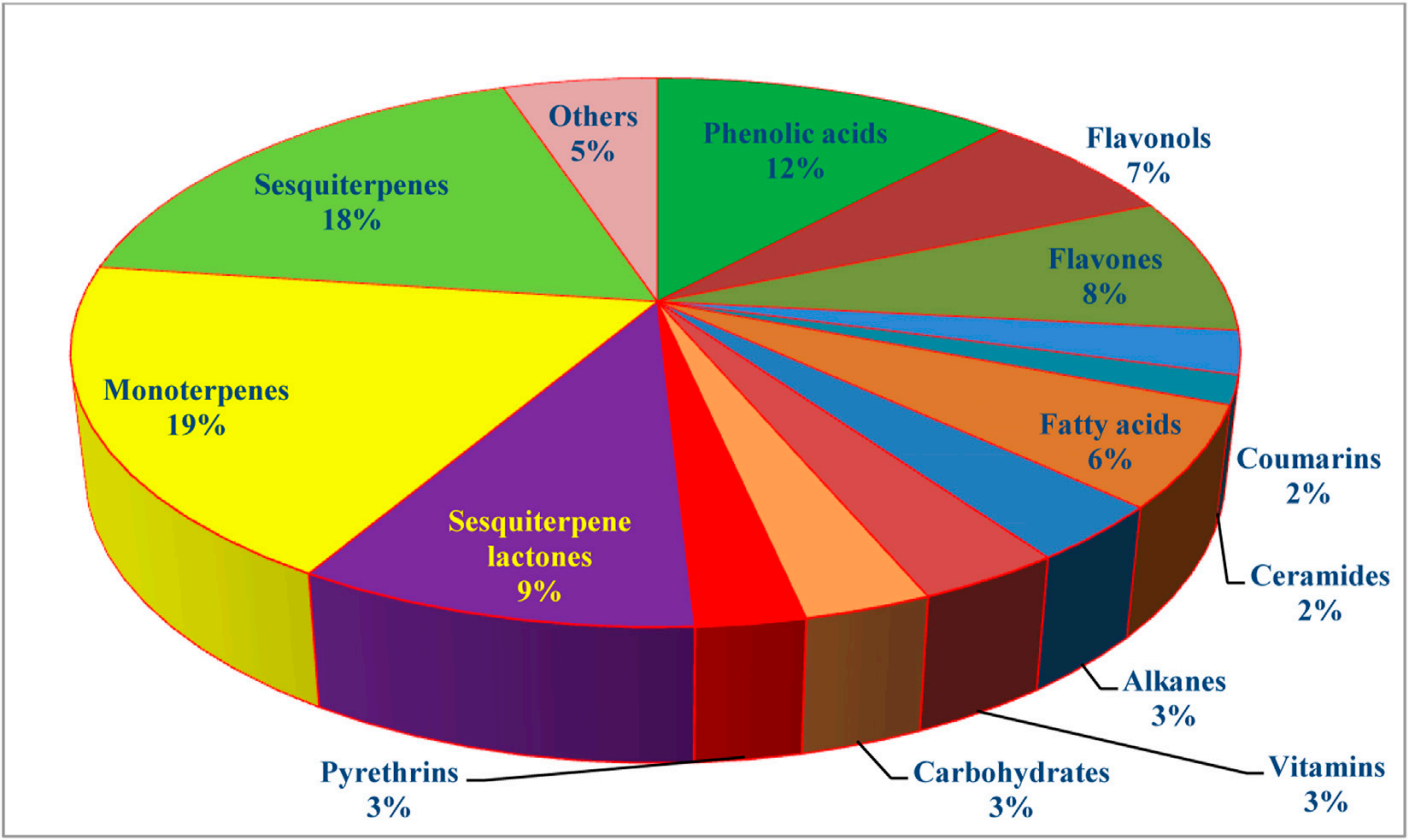
Isolated metabolites by chemical classes and proportions in the genus *Tanacetum*.

The genus *Tanacetum* was demonstrated to be a rich source of both secondary and primary metabolites with a broad spectrum of therapeutic merits. Analysis of more than 240 identified metabolites showed that monoterpenes are the preponderant metabolites **(19%)**, followed by sesquiterpenes **(18%)**, flavonoids **(15%)**, phenolic acids **(12%)**, and fatty acids and alkanes **(9%)** (Figure 5).

### 7.1 Phenolic acids

Phenolic acids are aromatic acids with a phenolic ring and at least a carboxylic functional group (Kumar and Goel, 2019). They are categorized into two main subclasses, namely, hydroxybenzoic acids and hydroxycinnamic acids (known as phenol carboxylic acids) (Kumar and Goel, 2019). So far, 28 phenolic acids have been identified in the *Tanacetum* species (**1-28**), including 22 hydroxycinnamic acids (**1-22**) and six hydroxybenzoic acids (**23-28**) (Benedec et al., 2016; Bączek et al., 2017; Devrnja et al., 2017; Rezaei et al., 2017). *T. vulgare* is the richest source of phenolic acids; eighteen phenolic acids (**1, 2, 4**, **5**, **10-23**, and **25**) have been successfully found and identified, predominantly from the leaves, flowers, aerial parts, and roots using mainly high-performance liquid chromatography (HPLC). These phenolic compounds are mainly derivatives of *p*-coumaric acid (**6**), caffeic acid (**2**), and ferulic acid (**7**). Moreover, Eight caffeoylquinic acid derivatives were identified in the aerial parts of two *Tanacetum* taxa using HPLC fingerprinting analysis, including one in *T. balsamita* (**3**) and seven in *T. vulgare* (**12-15, 18, 20, 22**) (Yu et al., 2017; Ak et al., 2021).

### 7.2 Flavonoids

Thirty-five flavonoids (**31-65**) have been isolated and identified from the aerial parts, leaves, and whole plants of *T. vulgare*, *T. balsamita*, *T. densum*, *T. cilicicum*, *T. parthenium*, *T. sinaicum*, *T. parthenifolium*, and *T. zahlbruckneri*. Flavonoids in the genus *Tanacetum* can be divided into two main subclasses according to their structural variations, namely, flavonols (**31-46**) and flavones (**47-65**). The name, species and parts sources, and skeleton types of these metabolites are listed in the (Table 3). Several studies correlated these secondary metabolites with the free radical scavenging capacity. For instance, the hydroethanolic extract from *T. balsamita* and *T. vulgare*-air dried whole plant displayed antioxidant capacity at DPPH and FRAP assays (IC_50_ = 13.59 ± 0.21 µmol Trolox/g extract, IC_50_ = 13.86 ± 0.19 µmol Trolox/g extract in DPPH assay, respectively, and IC_50_ = 339.1 ± 17.12 µmol Trolox/g extract, 585.6 ± 2.05 µmol Trolox/g extract in FRAP assay, respectively) (Bączek et al., 2017). The ethanolic extract from flowers and leaves of six Iranian *Tanacetum* taxa, namely, *T. tabrisianum*, *T. sonboli*, *T. chiliophyllum*, *T. hololeucum*, *T. kotschyi*, and *T. budjnurdense*, displayed *in vitro* antioxidant activity in the DPPH assay with IC_50_ values ranging from 59.55 to 157.24 µg/mL (Esmaeili et al., 2010). While these *in vitro* assays can provide preliminary information on the antioxidant capacity of a compound/extract, *in vivo* studies are necessary to fully evaluate their pharmacological relevance and explore their safety, efficacy, and potential mechanisms of action.

**TABLE 3:** Chemical compounds detected in the species of the genus *Tanacetum*.

Compound	Parts used	Solvent	Species	Analytic method	Country	References
**Hydroxycinnamic acid derivatives**						
Chlorogenic acid^**1**^	L, WP	MeOH	*T. vulgare*	HPLC-UV	Poland Türkiye	Bączek et al. (2017)
			*T. macrophyllum*			
			*T. corymbosum*			
			*T. balsamita*			
			*T. cilicicum*			
Caffeic acid^**2**^	WP, Fl	EtOH/W (40:60 v/v)70% EtOH MeOH	*T. vulgare*	HPLC, HPLC–MS	Poland Türkiye, Romania	Bączek et al. (2017)
			*T. balsamita*			
			*T. parthenium*			
3,5-*O*-Dicaffeoylquinic acid^**3**^	L	EtOH/W (40:60 v/v)	*T. balsamita*	HPLC-DAD		Pukalskas et al. (2010)
Rosmarinic acid^**4**^	WP	EtOH/W (40:60 v/v)	*T. vulgare*	HPLC	Poland, Türkiye	Bączek et al. (2017)
			*T. balsamita*			
Chicoric acid^**5**^	WP	EtOH/W (40:60 v/v)	*T. vulgare*	HPLC	Poland, Türkiye	Bączek et al. (2017)
			*T. balsamita*			
*p*-Coumaric acid^**6**^	AP	70% EtOH	*T. balsamita*	HPLC-MS	Iran	Benedec et al. (2016)
			*T. parthenium*			
Ferulic acid^**7**^	AP	70% EtOH MeOH	*T. balsamita*	HPLC-MS	Iran	Benedec et al. (2016), Rezaei et al. (2017)
			*T. parthenium*			
Cinnamic acid^**8**^	AP	EtOH	*T. balsamita*	HPLC	Türkiye	Savci et al. (2020)
			*T. chiliophyllum*			
			*T. zahlbruckneri*			
			*T. parthenifolium*			
Sinapic acid^**9**^	AP	MeOH	*T. parthenium*	HPLC	Iran	Rezaei et al. (2017)
Neochlorogenic acid^**10**^	Fl, R, L	MeOH	*T. vulgare*	LC-DAD/ESI-TOF-MS	Serbia	Devrnja et al. (2017)
Cryptochlorogenic acid^**11**^						
1-Caffeoylquinic acid^**12**^						
3,4-*O*-Dicaffeoylquinic acid^**13**^						
3,5-*O*-Dicaffeoylquinic acid^**14**^						
4,5-*O*-dicaffeoylquinic acid^**15**^						
3,5-Dichlorogenic acid^**16**^	Fl	70% EtOH	*T. vulgare*	HPLC	Ukraine	Yu et al. (2017)
4,5-Dichlorogenic acid^**17**^						
2-*O*-Caffeoylglucaric acid^**18**^	AP	H_2_O EtOH/H_2_O	*T. vulgare*	UHPLC-HRMS	Türkiye	Ak et al. (2021)
3-*O*-Feruloylquinic acid^**19**^							
1-Caffeoyl-3-hydroxy-dihydrocaffeoylquinic acid^**20**^							
Caffeic acid-*O*-hexoside^**21**^							
4,5-Dicaffeoylquinic acid^**22**^							
**Hydroxybenzoic acid derivatives**						
Gentisic acid^**23**^	AP, Fl	70% EtOH	*T. balsamita* ^**α**^, *T. vulgare* ^**β**^	HPLC-MS^**α**^, UHPLC-HRMS^**β**^	Romania^**α**^, Türkiye^**β**^	Benedec et al. (2016); Ak et al. (2021)
Salicylic acid^**24**^	AP	EtOH	*T. balsamita*	HPLC		Savci et al. (2020)
4-Hydroxybenzoic acid^**25**^	AP	EtOH	*T. balsamita*	HPLC	Türkiye, Iran	Rezaei et al. (2017); Savci et al. (2020)
			*T. parthenifolium*			
			*T. zahlbruckneri*			
			*T. chiliophyllum*			
			*T. parthenium, T. vulgare*			
Gallic acid^**26**^	AP	MeOH	*T. cilicicum*	HPLC-UV	Türkiye, Iran	Gecibesler et al. (2016), Rezaei et al. (2017)
			*T. parthenium*			
Syringic acid^**27**^	AP	MeOH	*T. cilicicum*	HPLC-UV	Turkey, Iran	Gecibesler et al. (2016), Rezaei et al. (2017)
			*T. parthenium*			
Vanillic acid^**28**^	AP	MeOH	*T. parthenium*	HPLC	Iran	Rezaei et al. (2017)
**Cyclitols**						
Shikimic acid^**29**^	AP	H_2_O, EtOH/H_2_O	*T. vulgare*	UHPLC-HRMS	Türkiye	Ak et al. (2021)
Quinic acid^**30**^						
**Flavonols**						
Quercetin^**31**^	AP	70% EtOH^**α**^, MeOH^**β**^	*T. balsamita* ^**α**^, *T. vulgare* ^**β**^	HPLC-MS	Romania	Benedec et al. (2016), Ivănescu et al. (2021)
Quercitrin^**32**^						
Isoquercitrin^**33**^						
Rutin^**34**^	AP^**α**^, WP^**β**^	70% EtOH^**α**^, Isopropanol/hexane (2:3 v/v)^**β**^	*T. balsamita* ^**α**^, *T. densum* ^**β**^	HPLC-MS	Romania^**α**^, Türkiye^**β**^	Benedec et al. (2016), Emre (2021)
Kaempferol^**35**^						
5,7,3',4'-Tetrahydroxy-3,8-dimethoxyflavonol^**36**^	AP	MeOH/W/HOAc (79:20:1)	*T. balsamita*	HPLC-DPPH	Lithuania	Pukalskas et al. (2010)
Catechin^**37**^	WP^**α,γ**^, AP^**β**^	Isopropanol/hexane (2:3 v/v)^**α**^,MeOH^**β,γ**^	*T. densum* ^**α**^	HPLC–UV	Türkiye^**α,β**^, Italy^**γ**^	Gecibesler et al. (2016), Emre (2021), Recinella et al. (2021)
			*T. cilicicum* ^**β**^			
			*T. parthenium* ^**γ**^			
Galangin^**38**^	AP	MeOH	*T. cilicicum*	HPLC–UV	Türkiye	Gecibesler et al. (2016)
6-Hydroxykaempferol^**39**^	L	80% MeOH	*T. vulgare, T. parthenium*	HPLC, NMR	Germany	Williams et al. (1999)
6-Hydroxykaempferol 3,5,7-trimethyl ether^**40**^						
Myricetin^**41**^	WP	80%MeOH^**α**^, EtOH^**β**^	*T. densum* ^**α**^	HPLC-DAD	Türkiye	Emre (2021)
			*T. balsamita* ^**β**^			
Jaceidin^**42**^	AP	H_2_Cl_2_/MeOH (1:1)	*T. sinaicum*	TLC, MS NMR	Egypt	Marzouk et al. (2016)
Chrysosplenetin^**43**^						
Vitexicarpin^**44**^						
Casticin^**45**^						
Centaureidin^**46**^	AP	EtOAc	*T. parthenium*	HPLC, NMR	France	Long et al. (2003)
**Flavones**						
Chrysin^**47**^	AP	MeOH	*T. parthenium*	HPLC–UV	Türkiye	Gecibesler et al. (2016)
Apigenin^**48**^	AP^**α**^, L^**γ**^	MeOH^**α**^, 80% MeOH^**γ**^, H_2_Cl_2_/MeOH (1:1)^**β**^	*T. macrophyllum* ^**α**^	HPLC,TLC, MS,NMR	Romania^**α**^, Egypt^**α,β**^, Germany^**γ**^	Williams et al. (1999); Marzouk et al. (2016), Ivănescu et al. (2021)
			*T. vulgare* ^**α**^			
			*T. corymbosum* ^**α**^			
			*T. parthenium* ^**γ**^			
			*T. sinaicum* ^**α,β**^			
Acacetin^**49**^	AP^**α**^, L^**β**^	H_2_Cl_2_/MeOH (1:1)^**α**^, MeOH^**β**^	*T. sinaicum* ^**α**^	LC-MS, TLC, MS, NMR	Egypt^**α**^, Japan^**β**^	Uehara et al. (2015), Marzouk et al. (2016)
			*T. vulgare* ^**β**^			
Luteolin^**50**^	AP	H_2_Cl_2_/MeOH (1:1)^**α**^, MeOH^**β**^, 80% EtOH^**γ**^	*T. sinaicum* ^**α**^	TLC, MS, NMR	Egypt^**α**^, United kingdom^**β**^, United States^**γ**^	Juan-Badaturuge et al. (2009), Marzouk et al. (2016)
			*T. vulgare* ^**β**^			
			*T. parthenium* ^**γ**^			
Cirsilineol^**51**^	AP^**α**^, WP^**β**^	H_2_Cl_2_/MeOH (1:1)^**α**^, MeOH^**β**^	*T. sinaicum* ^**α**^	TLC, MS, NMR	Egypt^**α**^, Türkiye^**β**^	Polatoğlu et al. (2013), Marzouk et al. (2016)
			*T. chiliophyllum* ^**β**^			
Santin^**52**^	AP	EtOAc	*T. parthenium*	HPLC, NMR	France	Long et al. (2003)
Apigenin 7-*O*-β-glucopyranoside^**53**^	AP^**α**^, L^**β**^	H_2_Cl_2_/MeOH (1:1)^**α**^, MeOH^**β**^	*T. sinaicum* ^**α**^, *T. vulgare* ^**β**^	TLC, LC-MS, MS, NMR	Egypt^**α**^, Finland^**β**^, Sweden^**β**^	Uehara et al. (2015), Marzouk et al. (2016)
Apigenin 7-*O*-β-glucuronide^**54**^						
Luteolin 7-*O*-β-glucopyranoside^**55**^						
luteolin 7-*O*-β-glucuronide^**56**^						
Chrysoeriol 7-*O*-glucuronide^**57**^	L	MeOH	*T. vulgare*	LC-MS, UV, HPLC	Japan	Uehara et al. (2015)
Hispidulin^**58**^						
Nepetin^**59**^						
Eupatilin^**60**^						
Jaceosidin^**61**^						
Pectolinarigenin^**62**^						
5,7,4'-trihydroxy-3,6-dimethoxyflavone^**63**^	AP	EtOH	*T. oshanahanii*	^1^H NMR, IR, ^13^C NMR	Spain	Triana et al. (2013)
5-hydroxy-3,6,7,8,3’,4’-hexamethoxyflavone^**64**^	WP	MeOH	*T. artemisioides*	2D-NMR, IR, COSY, HMQC	Pakistan	Hussain et al. (2010)
5-Demethylnobiletin^**65**^						
**Coumarins**						
Isofraxidin^**66**^	R	*n*-Hexane	*T. parthenium*	HPLC, NMR	Poland	Kisiel and Stojakowska (1997)
9-Epipectachol B^**67**^						
Scopoletin^**68**^	AP^**α,β**^, Sd^**γ**^, Pe^**δ**^	EtOAc^**α**^, EtOH^**β**^,CH_2_Cl_2_^**γ**^, EO^**δ**^	*T. cadmeum* ^**α**^	^1^H NMR, IR, UV, MS, GC-MS	Türkiye^**α**^, Spain^**β**^, United Kingdom^**γ**^, Iran^**δ**^	Gonzalez et al. (1990), Susurluk et al. (2007)
			*T. ferulaceum* ^**β**^			
			*T. parthenium* ^***γ***^			
			*T. balsamita* ^**δ**^			
Scoparone^**69**^	AP	EtOH	*T. ferulaceum*	^1^H NMR, IR, ^13^C NMR	Spain	Gonzalez et al. (1990), Triana et al. (2013)
			*T. ptarmiciflorum*			
			*T. oshanahanii*			
7-Hydroxycoumarin (Umbelliferone)^**70**^	AP	MeOH	*T. cadmeum*	X-ray, ^1^H NMR, IR, TLC	Türkiye	Çalişkan et al. (2004), Servi̇ and Gören (2019)
			*T. mucroniferum*			
Dimethylfraxetin (6,7,8-Trimethoxycoumarin)^**71**^	AP	EtOH	*T. ferulaceum*	^1^H NMR, MS	Spain	Gonzalez et al. (1990)
**Ceramides**						
Tanacetamide A^**72**^	WP	MeOH	*T. artemisioides*	2D-NMR, IR, COSY, HMQC	Pakistan	Ahmad et al. (2004)
Tanacetamide B^**73**^						
Tanacetamide C^**74**^	WP	MeOH	*T. artemisioides*	2D-NMR, IR, COSY, HMQC	Pakistan	Hussain et al. (2005)
Tanacetamide D^**75**^	WP	MeOH	*T. artemisioides*	2D-NMR, IR, COSY, HMQC	Pakistan	Hussain et al. (2010)
**Fatty acids**						
Capric acid^**76**^	AP^**α**^, St^**β**^ Fl^**β**^, WP^**γ**^	EO^**α**^ *n*-Hexane^**β**^ Isopropanol/Hexane (2:3 v/v)^**γ**^	*T. parthenium* ^**α**^, *T. zahlbruckneri* ^**β**^, *T. densum* ^**γ**^	GC-MS^**α,B**^ HPLC^**γ**^	Iran, Türkiye	Caglar et al. (2017), Rezaei et al. (2017); Emre (2021)
Myristic acid^**77**^						
Palmitic acid^**78**^						
Palmitoleic acid^**79**^						
Stearic acid^**80**^						
Oleic acid^**81**^						
Linoleic acid^**82**^						
Arachidic acid^**83**^						
Erucic acid^**84**^	AP	EO	*T. parthenium*	GC-MS	Iran	Rezaei et al. (2017)
Lignoceric acid^**85**^						
Lauric acid^**86**^						
Margaric acid^**87**^	R, Fl	*n*-Hexane	T. zahlbruckneri*	GC-MS	Iran	Caglar et al. (2017)
*cis*-Vaccenic acid^**88**^						
Behenic acid^**89**^						
**Alkanes**						
Octadecane^**90**^	Inf	EO	*T. vulgare*	GC-MS	Finland	Korpinen et al. (2021)
Eicosane^**91**^						
Docosane^**92**^						
n-Tricosane^**93**^						
Pentacosane^**94**^						
Heptacosane^**95**^						
*n*-Nonacosane^**96**^						
Hentriacontane^**97**^						
**Fat-soluble vitamins**						
Vitamin K1^**98**^	WP	i-PrOH/Hexane (2:3 v/v)	*T. densum*	HPLC	Türkiye	Emre (2021)
Vitamin K2^**99**^						
Vitamin D2^**100**^						
Vitamin D3^**101**^						
α-Tocopherol^**102**^						
β-Tocopherol^**103**^						
Retinol^**104**^						
Retinol acetate^**105**^						
**Carbohydrates**						
Glucose^**106**^	R, Fl	0.75% AmOx	*T. vulgare*	GC-MS, NMR	Russia	Polle et al. (2001)
Xylose^**107**^						
Mannose^**108**^						
Galactose^**109**^						
Arabinose^**110**^						
Apiose^**111**^						
2-*O*-methylxylose^**112**^						
**Pyrethrins**						
Pyrethrin I^**113**^	Fl	*n*-Hexane, Ace, PEE, i-PrOH, SFE	*T. cinerariifolium*	HPLC–UV, SFC-FID, RP-HPLC– DAD	Kenya, Croatia, India, Italy	Kasaj et al. (1999), Jeran et al. (2021)
Pyrethrin II^**114**^						
Cinerin I^**115**^						
Cinerin II^**116**^						
Jasmolin I^**117**^						
Jasmolin II^**118**^						
**Sesquiterpene lactones**						
Parthenolide^**119**^	AP	EtOH-H_2_O (90:10)	*T. parthenium*	LC-MS,^13^C NMR, ^1^H NMR	Brazil	Tiuman et al. (2005)
Costunolide^**120**^	AP	EtOH	*T. ferulaceum*	^1^H NMR, MS	Spain	Gonzalez et al. (1990)
Tatridin A^**121**^						
11,13-Dihydrotatridin A^**122**^						
Tatridin B^**123**^						
Spiciformin^**124**^						
lα,10β-Epoxydeacetyllaurenobiolide^**125**^						
Deacetyl-beta-cyclopyrethrosin^**126**^						
6α-Hydroxy-5,7αH,8βH-eudesm-4 (15)-en-8,12-olide^**127**^						
4β,6α-Dihydroxy-5,7αH,8βH-eudesman-8,12-olide^**128**^						
Douglanin^**129**^	Fl	PEE	*T. vulgare*	^13^C-NMR, ^1^H-NMR	Italy	Rosselli et al. (2012)
Ludovicin A^**130**^						
Ludovicin B^**131**^						
1α-Hydroxy-1-deoxoarglanine^**132**^						
11,13-Dehydrosantonin^**133**^						
A, β-Cyclopyrethrosin^**134**^	Fl	EtOH	*T. cinerariifolium*	CC, NMR	Canada	Doskotch et al. (1971)
Chrysanin^**135**^						
Pyrethrosin^**136**^						
Dehydro-β-cyclopyrethrosin^**137**^						
Chrysanolide^**138**^						
B, (11R)-11,13-Dehydro-tatridin-A^**139**^						
**Monoterpene hydrocarbons**						
α-Pinene^**140**^	WP^**α,β**^, L^**γ**^, AP^**δ,ζ**^, F^**ε**^	EO	*T. balsamita* ^***α***^ *, T. vulgare* ^**β**^, *T. parthenium* ^***γ***^, *T. sinaicum* ^**δ**^, *T. cilicicum* ^**ε**^, *T. chiliophyllum* ^**ζ**^, *T. densum* ^**ε**^	GC-FID, GC–MS	Italy^**α,β**^, Iran^**γ**^, Egypt^**δ**^, Türkiye^**ε, ζ**^	Polatoğlu et al. (2009), Bączek et al. (2017); Farzadfar et al. (2017); Elshamy et al. (2021)
β-Pinene**^141^**	WP^**α,β**^, L^**γ**^, AP^**δ, ζ**^	EO	*T. balsamita* ^***α***^ *, T. vulgare* ^**β**^, *T. parthenium* ^***γ***^, *T. sinaicum* ^**δ**^, *T. chiliophyllum* ^**ε**^, *T. chiliophyllum* ^**ζ**^	GC-FID, GC–MS	Italy^**α,β**^, Iran^**γ**^, Egypt^**δ**^, Türkiye^**ε, ζ**^	Salamci et al. (2007), Bączek et al. (2017), Farzadfar et al. (2017); Elshamy et al. (2021)
Sabinene**^142^**	WP^**α**^, F^**β,γ**^, L ^**γ**^	EO	*T. balsamita* ^***α*,β**^ *, T. vulgare* ^**α**^, *T. macrophyllum* ^**γ**^	GC-FID, GC–MS	Italy^**α**^, Iran^**β**^, Türkiye^**γ**^	Polatoğlu et al. (2015); Bączek et al. (2017); Farzadfar et al. (2017)
β-Myrcene**^143^**	AP, L, F	EO	*T. balsamita*	GC–MS	Iran	Monfared et al. (2002)
Limonene**^144^**	AP	EO	*T. balsamita*	GC–MS	Türkiye	Bagci et al. (2008)
Terpinolene**^145^**	AP^**α**^, WP^**β**^	EO	*T. chiliophyllum* ^**α**^, *T. balsamita* ^**β**^	GC–MS	Türkiye^**α**^, Italy^**β**^	Bagci et al. (2008), Bączek et al. (2017)
α-Thujene**^146^**	AP^**α, β**^, WP^**γ**^	EO	*T. chiliophyllum* ^**α**^, *T. cilicicum* ^**β**^, *T. balsamita* ^**γ**^, *T. vulgare* ^**γ**^	GC-FID	Türkiye^**α, β**^, Italy^**γ**^	Salamci et al. (2007), Bagci et al. (2008)
p-Cymene**^147^**	F^**α**^, L^**α**^, AP^**β**^, WP^**γ**^	EO	*T. polycephalum* ^**α**^, *T. argenteum* ^**β**^, *T. balsamita* ^**γ**^, *T. vulgare* ^**γ**^	GC–MS	Iran^**α**^, Türkiye^**β,γ**^	Nori-Shargh et al. (1999), Bączek et al. (2017)
Camphene**^148^**	AP^**α**^, F^**β**^, L^**β**^, F^**γ**^, St^**γ**^	EO	*T. chiliophyllum* ^**α**^, *T. balsamita* ^**α**^, *T.polycephalum* ^**β**^, *T. kotschyi* ^γ*^	GC–MS	Iran^**α, β**^, Türkiye^**γ**^	Nori-Shargh et al. (1999), Bagci et al. (2008), Polatoglu et al. (2011)
Verbenene**^149^**	AP^**α**^	EO	*T. chiliophyllum* ^**α**^, *T. balsamita* ^**α**^	GC–MS	Iran^**α**^	Bagci et al. (2008)
Tricyclene**^150^**	AP^**α**^	EO	*T. chiliophyllum* ^**α**^, *T. balsamita* ^**α**^	GC–MS	Iran^**α**^	Bagci et al. (2008)
α-Phellandrene^**151**^	WP	EO	*T. vulgare*	GC–MS	Italy	Bączek et al. (2017)
Santolinatriene**^152^**	L^**α**^, F^**α**^, AP^**β**^	EO	*T. argyrophyllum* ^**α**^, *T. praeteritum* ^**β**^, *T. santolinoides* ^**β**^	GC–MS	Türkiye	Gören et al. (2001), El-Shazly et al. (2002)
β-Phellandrene**^153^**	AP	EO	*T. praeteritum*	GC–MS	Türkiye	Gören et al. (2001)
**Oxygenated monoterpenes**						
1,8-Cineole**^154^**	L^**α**^, F^**α**^, AP^**β**^	EO	*T. argyrophyllum* ^**α**^, *T. praeteritum* ^**β**^, *T. argenteum* ^**α**^, *T. balsamita* ^**β**^, *T. chiliophyllum* ^**β**^, *T. polycephalum* ^**α**^, *T. cilicicum* ^**β**^	GC–MS	Türkiye, Iran	Nori-Shargh et al. (1999), Gören et al. (2001), Bagci et al. (2008)
Dehydro-1,8-cineole**^155^**	AP	EO	*T. argyrophyllum*, *T. praeteritum*	GC–MS	Türkiye	Gören et al. (2001)
1,4-Cineole**^156^**	WP	EO	*T. balsamita*	GC–MS	Italy	Bączek et al. (2017)
α-Thujone**^157^**	AP^**α**^, L^**β**^, WP^**γ**^	EO	*T. santolinoides* ^**α**^, *T. argyrophyllum* ^**β**^, *T. praeteritum* ^**α**^, *T. chiliophyllum* ^**α**^, *T. vulgare* ^**γ**^	GC–MS	Türkiye^**α, β**^, Italy^**γ**^	Gören et al. (2001), Salamci et al. (2007)
β-Thujone **^158^**	AP^**α**^, L^**β**^, WP^**γ**^	EO	*T. santolinoides* ^**α**^, *T. argyrophyllum* ^**β**^, *T. praeteritum* ^**α**^, *T. chiliophyllum* ^**α**^, *T. vulgare* ^**γ**^, *T. balsamita* ^***γ***^	GC–MS	Türkiye^**α, β**^, Italy^**γ**^	Gören et al. (2001), Salamci et al. (2007)
Camphor**^159^**	WP^**α**^, L^**β**^, AP^**γ**^	EO	*T. vulgare* ^**α**^, *T. argyrophyllum* ^**β**^, *T. praeteritum* ^**γ**^	GC–MS	Italy^**α**^, Türkiye^**β, γ**^	Gören et al. (2001), Bączek et al. (2017)
Carvone**^160^**	WP^**α**^, L^**β**^, F^**β**^	EO	*T. balsamita* ^**α**^, *T. vulgare* ^**α**^, *T. argyrophyllum* ^**β**^	GC–MS	Italy^**α**^, Türkiye^**β**^	Gören et al. (2001), Bączek et al. (2017)
cis-Dihydrocarvone**^161^**	WP	EO	*T. vulgare*	GC–MS	Italy	Bączek et al. (2017)
trans-Dihydrocarvone**^162^**	L^**α**^, F^**α**^, WP^**β**^	EO	*T. argyrophyllum* ^**α**^, *T. vulgare* ^**β**^	GC–MS	Türkiye^**α**^, Italy^**β**^	Gören et al. (2001), Bączek et al. (2017)
α-Terpineol**^163^**	AP^**α**^, L^**β**^, F^**β**^	EO	*T. santolinoides* ^**α**^, *T. argyrophyllum* ^**β**^	GC–MS	Egypt^**α**^, Türkiye^**β**^	Gören et al. (2001), El-Shazly et al. (2002)
Pinocarvone**^164^**	WP	EO	*T. balsamita*	GC–MS	Italy	Bączek et al. (2017)
Borneol**^165^**						
Eucalyptol**^166^**						
trans-Carveol**^167^**	AP	EO	*T. tabrisianum*	GC–MS	Iran	Habibi et al. (2007)
Dehydro-1,8-cineole**^168^**	AP	EO	*T. balsamita*	GC–MS	Iran	Monfared et al. (2002)
trans-carvyl acetate**^165^**						
cis-carvyl acetate**^166^**						
Lavandulol**^167^**	AP	EO	T. paradoxum*	GC–MS	Iran	Habibi et al. (2007)
Carvacrol**^168^**	WP	EO	*T. vulgare*	GC–MS	Italy	Bączek et al. (2017)
Thymol**^169^**	AP	EO	T. sinaicum*	GC–MS	Egypt	Elshamy et al. (2021)
Grandisol**^170^**						
Piperitone**^171^**						
Myrtenol**^172^**	WP	EO	*T. balsamita, T. vulgare*	GC–MS	Italy	Bączek et al. (2017)
cis-Piperitol**^173^**						
cis-Chrysanthenol**^174^**						
Dihydrocarveol**^175^**						
α-Campholenal**^176^**						
Filifolone**^177^**	AP	EO	*T. sinaicum*	GC–MS	Türkiye	Elshamy et al. (2021)
p-Menth-1-en-8-ol**^188^**						
Eugenol**^189^**	AP	EO	T. chiliophyllum*	GC–MS	Türkiye	Salamci et al. (2007)
**Sesquiterpenes hydrocarbons**						
Copaene**^190^**	AP	EO	T. cilicicum*, *T. sinaicum*	GC–MS	Türkiye	Gecibesler et al. (2016), Elshamy et al. (2021)
β-Humulene**^191^**						
α-Calacorene**^192^**	AP	EO	*T. balsamita, T. chiliophyllum*	GC–MS	Türkiye	Bagci et al. (2008)
β-Funebrene**^193^**						
Ledene**^194^**						
Bicyclogermacrene**^195^**						
Germacrene D**^196^**						
Ar-Curcumene**^197^**	AP	EO	T. chiliophyllum, T. aucherianum*	GC–MS	Türkiye	Salamci et al. (2007)
γ-Cadinene**^198^**						
δ-Cadinene**^199^**						
Allo-Aromadendrene**^200^**						
β-Farnesene**^201^**						
β-Caryophylene**^202^**						
β-Cubebene**^203^**						
Valeranone**^204^**	WP	EO	*T. balsamita, T. vulgare*	GC–MS	Italy	Bączek et al. (2017)
α-Longipinene**^205^**						
**Oxygenated sesquiterpenes**						
T-Muurolol**^206^**	AP	EO	*T. balsamita*	GC–MS	Iran	Monfared et al. (2002)
1,10-di-epi-Cubenol**^207^**						
β-Copaen-4-alpha-ol**^208^**						
Nerolidol**^209^**						
Cubebol**^210^**						
(Z)-Sesquilavandulol**^211^**						
trans-Isolongifolanone**^212^**	AP	EO	T. tabrisianum*	GC–MS	Iran	Habibi et al. (2007)
*Caryophyllene oxide* **^213^**						
*Davana ether-1* **^214^**	AP	EO	T. sinaicum*	GC–MS	Egypt	Elshamy et al. (2021)
Davana ether-2**^215^**						
Isoaromadendrene epoxide**^216^**						
Geranyl tiglate**^217^**						
Eudesm- 4(14)-en- 4-ol**^218^**						
Intermedeol**^219^**	WP	EO	*T. balsamita, T. vulgare*	GC–MS	Italy	Bączek et al. (2017)
β-Eudesmol**^220^**						
Longiverbenone**^221^**						
Acorenone B**^222^**						
Valeranone**^223^**						
Gleenol**^224^**						
Cadin-4-en-10-ol**^225^**						
β-Atlantone**^226^**	AP	EO	*T. balsamita, T. chiliophyllum*	GC–MS	Türkiye	Bagci et al. (2008)
Oplopenone**^227^**						
Ledol**^228^**						
Spathulenol**^229^**						
Isospathulenol**^230^**						
Hedycaryol**^231^**						
**Diterpenes**						
Neophytadiene**^232^**	AP	EO	*T. balsamita, T. chiliophyllum*	GC–MS	Türkiye	Bagci et al. (2008)
Vulgarol A**^233^**						
**α,β-Unsaturated aldehydes**						
(E)-2-Heptenal**^234^**	Fl	*n*-Hexane	*T. balsamita*	GC–MS	Brazil	Kubo and Kubo (1995)
(E)-2-Octenal**^235^**						
(E)-2-Nonenal**^236^**						
(E)-2-Decenal**^237^**						
(E)-2-Undecenal**^238^**						
(E,E)-2,4-Decadienal**^239^**						
3-Methyl-2-butenal**^240^**						
Hexanal**^241^**						

WP, whole plant; L, leaves; Fl, Flowers; St, Stems; Inf, Inflorescences; Pe, Petals; Flo: Floscules.

*T. aucherianum: Tanacetum aucherianum Sch.Bip.

*T. chiliophyllum: Tanacetum chiliophyllum var. chiliophyllum.

*T. sinaicum: Tanacetum sinaicum (Fresen.) Delile ex K.Bremer & Humphries.

*T. tabrisianum: Tanacetum tabrisianum (Boiss.) Sosn.& Takht.

*T. paradoxum: Tanacetum paradoxum Bornm.

*T. zahlbruckneri: T. zahlbruckneri (Nab.) Grierson.

*T. kotschyi: *T. kotschyi* (Boiss.) Grierson.

### 7.3 Coumarins

Coumarins are naturally occurring phenolic metabolites formed through condensing benzene and β-pyrone rings (Bouhaoui et al., 2021). These secondary compounds are categorized into four basic subgroups; simple coumarins, furanocoumarins, pyranocoumarins, and pyrone-substituted coumarins (4-Hydroxycoumarin, 3-phenylcoumarin, and 3,4-benzocoumarin) (Sarkhail, 2014). To date, 6 simple coumarins **(66-71)** have already been isolated and identified from the genus *Tanacetum* using HPLC, TLC, and spectroscopic methods, including NMR, UV, and IR, among others (Table 3). Kisiel and Stojakowski, (1997) have isolated and characterized isofraxidin (**66**) and 9-epipectachol B (**67**) from the hexane extract of *T. parthenium* roots (Kisiel and Stojakowska, 1997). Scopoletin (**68**) was detected in the aerial parts of four *Tanacetum* taxa, namely, *T. cadmeum*, *T. ferulaceum*, *T. parthenium*, and *T. balsamita* (Gonzalez et al., 1990; Susurluk et al., 2007). Scoparone (**69**) was found in the aerial parts methanolic extract of *T. ferulaceum* and *T. ptarmiciflorum*, while 7-hydroxycoumarin (**70**) was yielded from *T. cadmeum* and *T. mucroniferum* (Çalişkan et al., 2004; Triana et al., 2013; Servi̇ and Gören, 2019). Likewise, dimethylfraxetin (**71**) was reported in the ethanolic extract of *T. ferulaceum* aboveground parts (Gonzalez et al., 1990).

### 7.4 Ceramides

Ceramides are bioactive lipids made up of sphingosine and a fatty acid. They are abundantly found throughout chloroplast membranes and are crucial to biological processes, including apoptosis, cell senescence, differentiation, and stresses (Kurz et al., 2019). Indeed, *T. artemisioides* is almost the only species from the genus *Tanacetum* that have demonstrated to contain ceramides. Tanacetamides A and B **(72, 73)** were isolated and structurally elucidated from the chloroform soluble fraction of the whole plant methanolic extract based on 1D and 2D NMR analysis. In the same study, tanacetamides A and B disclosed substantial *in vitro* acetylcholinesterase inhibitory properties, with IC_50_ values of 67.1 ± 1.5 and 74.1 ± 5.0 μM, respectively, compared to the standard drug galanthamine (IC_50_ = 8.5 ± 0.0001 μM) (Ahmad et al., 2004). Likewise, tanacetamides C and D **(73, 74)**, with promising vasorelaxant properties, were isolated and identified from the chloroform fraction of the whole plant methanolic extract (Hussain et al., 2005; Hussain et al., 2010). However, the antihypertensive properties of these ceramides are still poorly understood. Thus, further *in vitro* and *in vivo* studies are required to corroborate the vasorelaxant properties of these compounds in line with the traditional usage of *T. artemisioides* as an antihypertensive agent.

### 7.5 Fatty acids and alkanes

Phytochemical investigations of three *Tanacetum* taxa have led to the isolation and identification of fourteen fatty acids. Nine saturated fatty acids (**76-78, 80, 83, 85-87,** and **89**) and five mono-and polyunsaturated fatty acids (**79, 81, 82, 84,** and **88**) were detected in the aerial parts, leaves, and flowers of *T. parthenium*, *T. zahlbruckneri*, and *T. densum*, using GC-MS and HPLC (Table 3) (Caglar et al., 2017; Rezaei et al., 2017; Emre, 2021). On the other hand, Korpinen et al. (2021) identified eight alkanes (**90-97**) from *T. vulgare* inflorescences essential oil based on the GC-MS analysis (Korpinen et al., 2021).

### 7.6 Pyrethrins

In southern Europe, the leaf decoction of several *Tanacetum* species, such as *T. cinerariifolium*, *T. vulgare* and *T. balsamita*, had been traditionally used as an insect repellent for cattle and children, and as household insecticides to control fleas and body lice (Cumo, 2013; Jeran et al., 2021; Souto et al., 2021). As early as the middle 19th century, the insecticidal properties of Pyrethrum, a natural extract retrieved from *T. cinerariifolium* flowers, have been widely recognized in the United States and Western Europe. By the early 20th century, Pyrethrum was already used to prevent insect-borne diseases (Malaria, leishmaniasis, and yellow fever, among others) and as an efficient alternative to synthetic pesticides due to its specific effect on target insects, short environmental lifespan (Half-live ranging from 2 h to 2 days), and limited mammalian toxicity (Cumo, 2013; Matsuo, 2019; Lybrand et al., 2020; Souto et al., 2021).

After being harvested, the plant’s flowers are reduced into a powder and then subjected to extraction with organic solvents, such as hexane and petroleum ether (Jeran et al., 2021). After the solvent removal, the active ingredients are recovered as an orange-colored liquid containing six naturally occurring insecticides called pyrethrins (Isman, 2006; Jeran et al., 2021). These metabolites have been identified/quantified as pyrethrin I and II, cinerin I and II, and jasmolin I and II (**113-118**) using mainly liquid chromatography coupled to UV or DAD detectors (Nagar et al., 2015; Jeran et al., 2021).

### 7.7 Dietary components

Polle et al. (2001) analyzed and quantified the polysaccharide contents in the roots, sprouts, and floscules of *T. vulgare* using aqueous ammonium oxalate extraction. They noted the presence of rhamnose, galactose, galacturonic acid, and arabinose residues as the main constituents, whereas glucose, mannose, apiose,2-*O*-methylxylose, and xylose residues were found in traces (Table 3) (Polle et al., 2001).

Analysis of fat-soluble vitamin contents in two *T. densum* subspecies (*T. densum* subsp. *laxum* and *T.* densum subsp. subsp. *amani*) revealed the presence of two forms of vitamin K, namely, vitamin K1 (1.5 ± 0.22 and 0.75 ± 0.19 μg/g, respectively) and vitamin K2 (traces).In addition, two forms of vitamin D (Vitamin D2 and D3), vitamin E (α-tocopherol and β-tocopherol), and vitamin A (Retinol and Retinol acetate) have also been alarmed in the two subspecies (0.05 ± 0.01, 0.05 ± 0.01; 0.1 ± 0.01, 0.2 ± 0.01; 7.3 ± 0.67, 5 ± 0.57; 0.55 ± 0.1, 0.8 ± 0.14; traces, 0.6 ± 0.1, 0.65 ± 0.26 μg/g, respectively) (Emre, 2021).

### 7.8 Sesquiterpene lactones

A total of 21 sesquiterpene lactones have been identified in the genus *Tanacetum*, including 8 germacranolide-type sesquiterpene lactones **(119-124, 138,** and **139)** and 13 eudesmane-type sesquiterpene lactones **(125-137)** (Table 3). Parthenolide **(119)** was isolated from the hydroethanolic extract of *T. parthenium* aerial parts (Tiuman et al., 2005). Moreover, the sesquiterpene lactones **(120-128)** were detected in the aerial parts ethanolic extract of *T. ferulaceum*. The metabolites **(129-133)** were found in a petroleum ether extract of *T. vulgare* flowers, whereas the sesquiterpenes **(133-139)** were yielded from flowers’ alcoholic extract of *T. cinerariifolium* (Gonzalez et al., 1990; Rosselli et al., 2012). These metabolites displayed potent antimicrobial, antioxidant, anticancer, anti-inflammatory, and neuroprotective activities (Fischedick et al., 2012; Rosselli et al., 2012).

### 7.9 Essential oil

The genus *Tanacetum* is a well-known source of essential oils (EOs) retrieved from various parts, especially aerial parts such as leaves, stems, and flowers using conventional hydrodistillation techniques such as Clevenger-type apparatus and advanced extraction techniques, including microwave-assisted extraction. The EO yields varied considerably between 0.04%–1.09% (v/w), depending on the species, extracted parts, and abiotic and biotic factors (Başer et al., 2001; El-Shazly et al., 2002; Salamci et al., 2007; Elshamy et al., 2021). The volatile constituents have been analyzed and quantified using GC-MS and GC-FID analyses. As such, a wide variety of chemical compounds belonging to diverse groups have been identified. These metabolites are mainly monoterpene hydrocarbons **(140-153)**, oxygenated monoterpenes **(154-189)**, sesquiterpenes hydrocarbons **(190-205)**, oxygenated sesquiterpenes **(206-231)**, and diterpenes **(232,** and **233)** (Table 3).

### 7.10 Other metabolites

Two cyclitols **(29, 30)** were detected in a hydroethanolic extract of the aerial parts of *T. vulgare* (Ak et al., 2021). Moreover, Kubo and Kubo, (1995) have isolated and identified eight α,β-unsaturated aldehydes **(234-241)** from an hexanic extract of *T. balsamita* flowers (Kubo and Kubo, 1995).

## 8 Biological activities

### 8.1 Antidiabetic activity

Carbohydrates are the primary constituents of the human diet occurring in panoply of beverages and foods. These hydrocarbons in the form of sucrose, starches, and fibers are broken down into glucose, which is subsequently absorbed, causing spikes in the systemic glycemia (Prasad et al., 2019). The cleavage of these macromolecules is under the control of key enzymes involved in carbohydrate digestion, such as α-glucosidase, β-glucosidase, and α-amylase (Al-Zuhair et al., 2010; Ramzi and Hosseininaveh, 2010; Olvera-Sandoval et al., 2022). Thereby, inhibiting or slowing down the activity of these target enzymes may effectively reduce the postprandial hyperglycemia and, therefore, successfully contribute to the management of diabetes mellitus (Khatib et al., 2022a).

In this sense, Özek (2018) evaluated the *in vitro* α-amylase inhibitory effects of *T. praeteritum* ssp. *praeteritum* aerial parts essential oils using the Caraway Somogyi iodine/potassium iodide (IKI) method and acarbose as the reference drug. The author indicated that the essential oil displayed α-amylase inhibitory features with an IC_50_ value of 0.89 ± 0.13 mg/mL compared to acarbose 0.08 mg/mL. The author attributed the inhibitory effects to the high amount of oxygenated monoterpenes in the EO (Özek, 2018).

Similarly, *T. haussknechtii* leaves, stems, and capitula essential oils and extracts (Methanol, water, and ethyl acetate), were *in vitro* assessed by Yur et al. (2017) for their α-amylase inhibitory capacities using the same method (I/KI). The authors observed that the water extracts had no inhibitory action on α-amylase, while the essential oils, methanol, and ethyl acetate extracts exhibited strong activity, with capitula ethyl acetate extract being the most active (356.9 ± 0.06 mg acarbose equivalent/g extract). The noticeable inhibitory effects of ethyl acetate extract were ascribed to the presence of caffeoylquinic acid derivatives endowed with substantial antidiabetic properties such as 1,3-*O*-dicaffeoylquinic acid, 3,4-*O*-dicaffeoylquinic acid, and 4,5-*O*-dicaffeoylquinic acid (Yur et al., 2017). *T. balsamita* aerial parts ethyl acetate extract displayed moderate inhibitory activity towards α-glucosidase enzyme with an IC_50_ value of 0.808 mg/mL. In a recent study, Gevrenova et al. (2023) reported that roots methanolic extract of *T. balsamita* had good α-glucosidase and α-amylase inhibitory effects with IC_50_ values of 0.71 ± 0.07 mmol acarbose/g and 0.43 ± 0.02 mmol acarbose/g, respectively (Gevrenova et al., 2023). However, no further *in vivo* studies have been carried out to assess the antidiabetic activity of *T. balsamita* extracts.

Furthermore, Khan et al. (2018) reported the capacity of *T. nubigenum* leaves ethanol extract and its butanol fraction at the concentrations of 10 μg/mL and 20 μg/mL to significantly inhibit human recombinant protein tyrosine phosphatase-1B (PTP-1B) up to 63.8%. In the same study, both ethanol and butanol extracts at 10 μg/mL substantially increased the glucose uptake in C2Cl2 cells by 61.2% and 41.2%, respectively (Khan et al., 2018).

On the other hand, the ethanol extract from *T. nubigenum* leaves at 60 mg/kg of body weight significantly dropped blood glucose level in STZ-induced Sprague-Dawley rats, after 5 h and 24 h by 15.5% and 10.8%, respectively, compared to the standard drug metformin (27.8%, 26.8%, respectively). The butanol fraction from the ethanolic extract showed stronger effects, decreasing the blood glucose levels by 17.9% and 21.3% after 5 h and 24 h, respectively (Khan et al., 2018).

**FIGURE 6:**
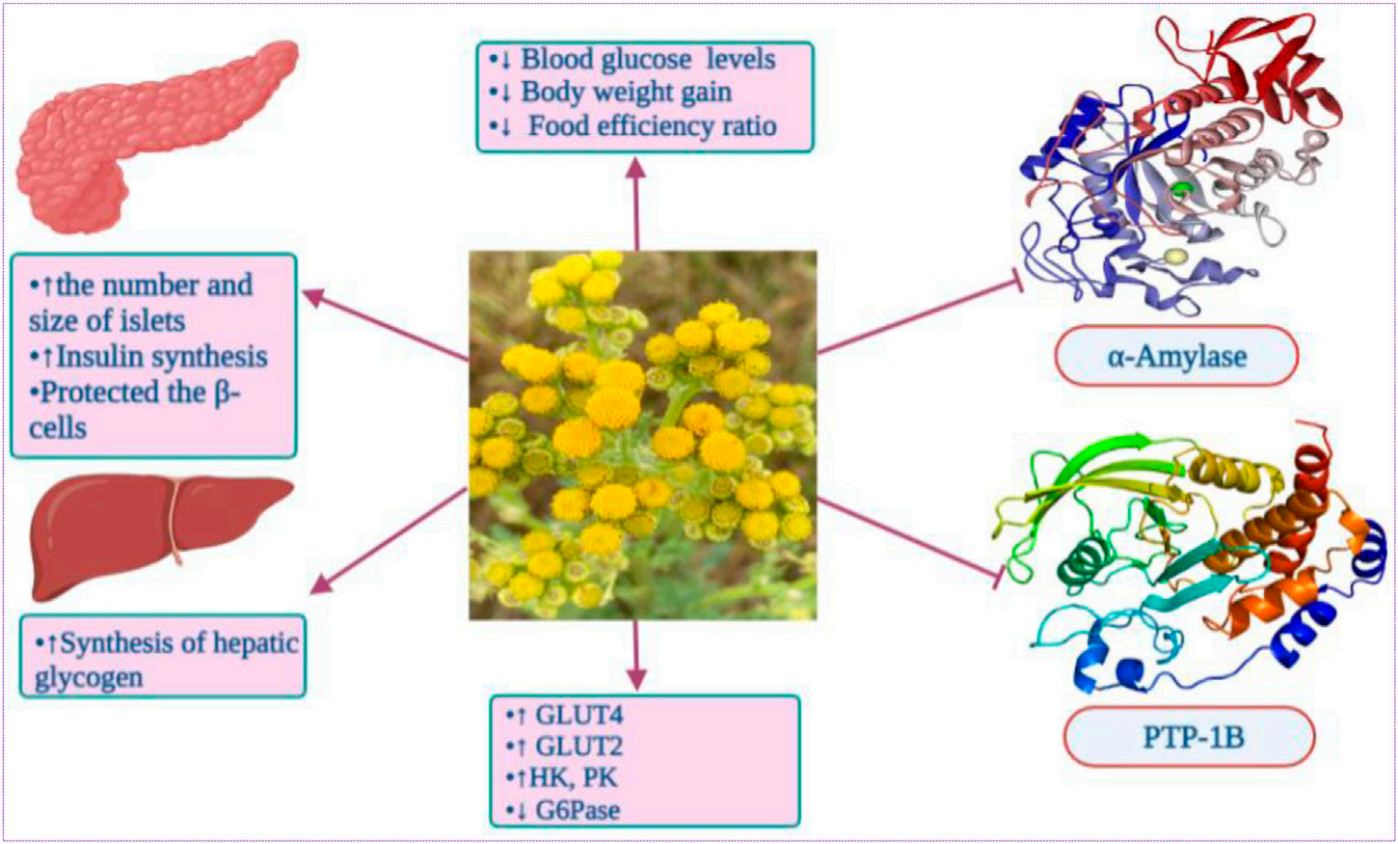
Potential antidiabetic action of *Tanacetum* spp. 2023^**©**^.

The antidiabetic action of *Tanacetum* spp. could be attributed to a myriad of active compounds, especially sesquiterpene lactones and phenolic compounds. For instance, parthenolide (**119**) from *T. parthenium* suppressed high-glucose stimulating IκBα protein degradation, nuclear factor kappa B (NF-κB) activation, growth factor beta (TGF-β1) and chemoattractant protein-1 (MCP-1) in mesangial cells (MCs) from rats (Jia et al., 2013). Chlorogenic acid, also known as 5-caffeoylquinic acid, has been identified in several *Tanacetum* leaves and whole plant. Previous clinical trials reported the ability of this phenolic acid to markedly reduce fasting blood glucose when consumed three times a day for 12 weeks at a dose of 400 mg capsules. Chlorogenic acid can improve glucose homeostasis by up-regulating the expression and translocation of glucose transporter type 4 (GLUT-4) in the skeletal muscle of mice models (Figure 6) (Wan et al., 2013). It has also been demonstrated to reduce the expression of serum vascular endothelial growth factor (VEGF)-mediating diabetic retinopathy in mice (Zhou et al., 2016).

However, only four taxa have been evaluated for their *in vitro* antidiabetic activity, namely, *T. praeteritum*, *T. haussknechtii*, *T. balsamita*, and *T. nubigenum*. Moreover, *T. nubigenum* is the only species assessed for its *in vivo* antidiabetic activity. We also noticed that all the *in vitro* studies are conducted using the caraway–Somogyi method. Therefore, using the 3,5-dinitrosalicylic acid reagent (DNS) method is recommended due to its ten-time sensitivity compared to the caraway-Somogyi method (Godinho et al., 2014). Likewise, it does not require stoichiometric data and allows comparing both methods. Additionally, further *in vivo* studies are needed to confirm the *in vitro* results.

### 8.2 Antimicrobial activity

*Tanacetum* species have been widely used for oral hygiene, festering wounds, skin ulcers and contusions, gastroenteritis, and venereal conditions. As demonstrated in (Table 4), the ethnomedicinal application of the plant as a traditional antimicrobial agent have been substantiated by several studies, especially crude extracts, essential oils, and isolated compounds. The antimicrobial potency of ethyl acetate extract (EtOAc) from air-dried aerial parts of *T. vulgare* was investigated against the caries-inducing fungus *C. albicans* (ATTC 1023) using the disc diffusion method (Kameri et al., 2019). The EtOAc alone disclosed moderate antifungal activity toward *C. albicans* after 24 h (IZD = 20 mm) at a dose of 100 mg/mL. The effect was more pronounced when supplementing the extract (100 mg/mL) with 2% chlorhexidine (IZD values ranging from 30 to 32.7 mm, after 5 min, 60 min, and 24 h, suggesting a synergetic effect toward *C. albicans* (Kameri et al., 2019). Pieces of evidence from a previous study depicted the efficacy of *T. balsamita* aerial parts EO against dental decay-causing bacteria, including *Streptococcus mutans* (PTCC 1683), *Streptococcus salivarius* (PTCC 1448), and *Streptococcus sanguinis* (PTCC 1449), compared to chlorhexidine and Oral B mouthwashes (Karimzadeh et al., 2021). The previous findings partially support the empirical usage of *Tanacetum* for dental hygiene, urging that the crude extracts and bioactive constituents could serve as targets for discovering new endodontic therapies.

**TABLE 4:** Antimicrobial properties of *Tanacetum* species.

Plant part	Extract/compound	Microbial strains	Method	Standard drug	Key results	References
*T. balsamita*						
WP	EO	*B. cereus* (ATCC 11778)	Serial microdilutions	Nr	MIC = 2 µL/mL	Bączek et al. (2017)
		*B. cereus* (15)			MIC = 2 µL/mL	
		*B. cereus* (X-13)			MIC = 4 µL/mL	
		*B. subtilis* (ATCC 6633)			MIC = 8 µL/mL	
		*S. aureus* (ATCC 25923)			MIC = 1 µL/mL	
		*S. aureus* (A-529)			MIC = 4 µg/mL	
		*S. epidermidis* (ATCC 12228)			MIC = 1 µL/mL	
		*L. monocytogenes* (17/11)			MIC = 32 µL/mL	
		*E. aerogenes* (ATCC 13048)			MIC = 8 µL/mL	
		*E. coli* (ATCC 25922)			MIC = 1 µL/mL	
		*E. coli* (O26 152/11)			MIC = 8 µg/mL	
		*K. pneumoniae* (ATCC 13883)			MIC = 8 µL/mL	
		*P. mirabilis* (ATCC 35659)			MIC = 2 µL/mL	
		*Y. enterocolitica* (O3 383/11)			MIC = 1 µL/mL	
		*P. aeruginosa* (ATCC 27853)			MIC = 32 µL/mL	
WP	EtOH/H_2_O (40:60, v/v)	*B. cereus* (ATCC 11778)	Serial microdilutions	Nr	MIC = 4 mg/mL	
		*B. cereus* (15)			MIC = 4 mg/mL	
		*B. cereus* (X-13)			MIC = 4 mg/mL	
		*B. subtilis* (ATCC 6633)			MIC = 4 mg/mL	
		*S. aureus* (ATCC 25923)			MIC = 2 mg/mL	
		*S. aureus* (A-529)			MIC = 2 mg/mL	
		*S. epidermidis* (ATCC 12228)			MIC = 1 mg/mL	
		*L. monocytogenes* (17/11)			MIC = 64 mg/mL	
		*E. aerogenes* (ATCC 13048)			MIC = 64 mg/mL	
		*E. coli* (ATCC 25922)			MIC = 64 mg/mL	
		*E. coli* (O26 152/11)			MIC = 64 mg/mL	
		*K. pneumoniae* (ATCC 13883)			MIC = 2 mg/mL	
		*P. mirabilis* (ATCC 35659)			MIC = 32 mg/mL	
		*Y. enterocolitica* (O3 383/11)			MIC = 2 mg/mL	
		*P. aeruginosa* (ATCC 27853)			MIC = 16 mg/mL	
AP	EO	*B. subtilis* (ATCC 465)	Disk diffusion	Amicasine 30E and Penicillin 10	The EO displayed moderate to significant antibacterial activity, with *S. epidermidis*, *B. pumulis*, and *B. subtilis* being the most prone to the EO (IZ = 35 ± 0.5, 34 ± 0.2, and 31 ± 0.3 mm, respectively, and MIC values of 0.93, 0.93 and 1.87 mg/mL, respectively)	Yousefzadi et al. (2009)
		*B. pumulis* (PTCC 1274)				
		*Enterococcus faecalis* (ATCC 29737)				
		*S. aureus* (ATCC 25923)				
		*S. epidermidis* (ATCC 12228)				
		*E. coli* (ATCC 25922)				
		*K. pneumoniae* (ATCC 10031)				
		*S. aureus* (ATCC 6538 P)				
		*B. cereus* (ATCC 14579)				
		*E. coli* (FV 755-0139)				
		*P. aeruginosa* (ATCC 27853)				
		*S. typhimurium* (ATCC 14028)				
***T. vulgare***						
WP	MeOH	*S. aureus* (ATCC 25923)	Disk diffusion	Ciprofloxacin	IZD = 16.5 ± 0.50 mm	Ivănescu et al. (2021)
		*E. coli* (ATCC 25922)			IZD = −	
		*P. aeruginosa* (ATCC 27853)			IZD = −	
		*C. albicans* (ATCC 90028)			IZD = 12.0 mm	
		*C. parapsilosis* (ATCC 22019)			IZD = 12.0 mm	
AP	EO	*E. coli* (ATCC 25922)			IC_50_ = 241 ± 13 µg/mL	
		*S. aureus* (ATCC 25923)			IC_50_ = 59 ± 5 µg/mL	
AP	1,8-Cineole	*E. coli* (ATCC 25922)	Disc diffusion and broth microdilution methods	Gentamycin and Chloramphenicol	IC_50_ > 200 µg/mL	Coté et al. (2017)
		*S. aureus* (ATCC 25923)			IC_50_ > 200 µg/mL	
AP	α-Humulene	*E. coli* (ATCC 25922)			IC_50_ > 200 µg/mL	
		*S. aureus* (ATCC 25923)			IC_50_ > 200 µg/mL	
AP	α-Terpinene	*E. coli* (ATCC 25922)			IC_50_ > 200 µg/mL	
		*S. aureus* (ATCC 25923)			IC_50_ > 200 µg/mL	
AP	β-Caryophyllene	*E. coli* (ATCC 25922)			IC_50_ > 200 µg/mL	
		*S. aureus* (ATCC 25923)			IC_50_ > 200 µg/mL	
AP	β-Pinene	*E. coli* (ATCC 25922)			IC_50_ > 200 µg/mL	
		*S. aureus* (ATCC 25923)			IC_50_ > 200 µg/mL	
AP	γ-Terpinene	*E. coli* (ATCC 25922)			IC_50_ > 200 µg/mL	
		*S. aureus* (ATCC 25923)			IC_50_ = 50 ± 9 µg/mL	
AP	Caryophyllene oxide	*E. coli* (ATCC 25922)			IC_50_ = 97 ± 2 µg/mL	
		*S. aureus* (ATCC 25923)			IC_50_ = 10.4 ± 0.9 µg/mL	
AP	Camphor	*E. coli* (ATCC 25922)			IC_50_ = 22 ± 1 µg/mL	
		*S. aureus* (ATCC 25923)			IC_50_ = 26 ± 3 µg/mL	
WP	EO	*B. cereus* (ATCC 11778)	Serial microdilutions	Nr	MIC = 1 µL/mL	Bączek et al. (2017)
		*B. cereus* (15)			MIC = 1 µL/mL	
		*B. cereus* (X-13)			MIC = 1 µL/mL	
		*B. subtilis* (ATCC 6633)			MIC = 4 µL/mL	
		*S. aureus* (ATCC 25923)			MIC = 2 µL/mL	
		*S. aureus* (A-529)			MIC = 8 µL/mL	
		*S. epidermidis* (ATCC 12228)			MIC = 0.5 µL/mL	
		*L. monocytogenes* (17/11)			MIC> 32 µL/mL	
		*E. aerogenes* (ATCC 13048)			MIC = 4 µL/mL	
		*E. coli* (ATCC 25922)			MIC = 8 µL/mL	
		*E. coli* (O26 152/11)			MIC> 32 µL/mL	
		*K. pneumoniae* (ATCC 13883)			MIC> 32 µL/mL	
		*P. mirabilis* (ATCC 35659)			MIC> 32 µL/mL	
		*Y. enterocolitica* (O3 383/11)			MIC = 1 µL/mL	
		*P. aeruginosa* (ATCC 27853)			MIC> 32 µL/mL	
WP	EtOH/H_2_O (40:60, v/v)	*B. cereus* (ATCC 11778)	Serial microdilutions	Nr	MIC = 8 mg/mL	
		*B. cereus* (15)			MIC = 8 mg/mL	
		*B. cereus* (X-13)			MIC = 8 mg/mL	
		*B. subtilis* (ATCC 6633)			MIC = 16 mg/mL	
		*S. aureus* (ATCC 25923)			MIC = 4 mg/mL	
		*S. aureus* (A-529)			MIC = 4 mg/mL	
		*S. epidermidis* (ATCC 12228)			MIC = 4 mg/mL	
		*L. monocytogenes* (17/11)			MIC = 64 mg/mL	
		*E. aerogenes* (ATCC 13048)			MIC> 64 mg/mL	
		*E. coli* (ATCC 25922)			MIC = 64 mg/mL	
		*E. coli* (O26 152/11)			MIC = 64 mg/mL	
		*K. pneumoniae* (ATCC 13883)			MIC = 4 mg/mL	
		*P. mirabilis* (ATCC 35659)			MIC = 16 mg/mL	
		*Y. enterocolitica* (O3 383/11)			MIC = 2 mg/mL	
		*P. aeruginosa* (ATCC 27853)			MIC = 32 mg/mL	
***T. parthenium***						
Fl, St	EO	*S. aureus* (ATCC 6538)	Broth microdilution	Chloramphenicol	IC_50_ = 250 µg/mL	Polatoglu et al. (2010)
		*S. epidermis* (ATCC 12228)			IC_50_ = 500 µg/mL	
		*B. cereus* (NRRL B-3711)			IC_50_ = 500 µg/mL	
		*B. subtilis* (NRRL B-437)			IC_50_ = 125 µg/mL	
		*E. coli* (NRRL B-3008)			IC_50_ = 500 µg/mL	
		*P. aeruginosa* (ATCC 27853)			IC_50_ = 500 µg/mL	
		*E. aerogenes* (NRRL 3567)			IC_50_ > 500 µg/mL	
		*P. vulgaris* (NRRL B-123)			IC_50_ > 500 µg/mL	
		*S. typhimurium* (ATCC 1331)			IC_50_ = 500 µg/mL	
AP (Fl)	EO (flowering)	*C. amalonaticus* (Lio)	Disk diffusion	Chloramphenicol	IZD = 16.5 ± 2 mm	Mohsenzadeh et al. (2011)
		*P. vulgaris* (Lio)			IZD = 21 ± 5 mm	
		*S. marcescens* (PTCC 1330)			IZD = 18 ± 4 mm	
		*E. aerugenes* (PTCC 10009)			IZD = 17 ± 3.5 mm	
		*B. cereus* (ATCC 7064)			IZD = 18 ± 2 mm	
		*B. megaterium* (PTCC 1672)			IZD = 29 ± 6 mm	
		*S. aureus* (ATCC 6633)			IZD = 24 ± 4 mm	
AP	EO (Pre-flowering)	*C. amalonaticus* (Lio)	Disk diffusion	Chloramphenicol	IZD = 15 ± 3 mm	
		*P. vulgaris* (Lio)			IZD = 16 ± 4 mm	
		*S. marcescens* (PTCC 1330)			IZD = 14 ± 2 mm	
		*E. aerugenes* (PTCC 10009)			IZD = 13 ± 3 mm	
		*B. cereus* (ATCC 7064)			IZD = 14 ± 2 mm	
		*B. megaterium* (PTCC 1672)			IZD = 25 ± 3 mm	
		*S. aureus* (ATCC 6633)			IZD = 18 ± 2.5 mm	
AP	EO (Post-flowering)	*C. amalonaticus* (Lio)	Disk diffusion	Chloramphenicol	IZD = 13 ± 3.5 mm	
		*P. vulgaris* (Lio)			IZD = 17 ± 4 mm	
		*S. marcescens* (PTCC 1330)			IZD = 13 ± 2 mm	
		*E. aerugenes* (PTCC 10009)			IZD = 13 ± 4 mm	
		*B. cereus* (ATCC 7064)			IZD = 15 ± 4 mm	
		*B. megaterium* (PTCC 1672)			IZD = 26 ± 3.5 mm	
		*S. aureus* (ATCC 6633)			IZD = 22 ± 4 mm	
***T. hololeucum (*Bornm.) Podlech**						
AP	EO	*S. aureus* (ATCC 25923)	Disk diffusion	Cefexime	IZD = 35 mm; MIC = 2 mg/mL	Shamkhani et al. (2016)
		*B. cereus* (PTCC 1015)			IZD = 14 mm; MIC = 2 mg/mL	
		*E. coli* (ATCC 25922)			IZD = 12 mm; MIC = 16 mg/mL	
		*K. Pneumoniae* (Clinical isolate)			IZD = 9 mm; MIC = 16 mg/mL	
***T. polycephalum***						
AP	EO	*T. harizanum*	Broth microdilution	Amphotericin B	MIC = 312.5 µg/mL; MFC = 312.5 µg/mL	Eblaghi et al. (2016)
		*B. spectabilis*			MIC = 625.0 µg/mL; MFC = 625.0 µg/mL	
		*P. variotii*			MIC = 312.5 µg/mL; MFC = 312.5 µg/mL	
		*P. chrysogenum*			MIC = 1250.0 µg/mL; MFC = 1250.0 µg/mL	
		*A. oryzae*			MIC = 625.0 µg/mL; MFC = 625.0 µg/mL	
		*A. niger*			MIC = 1250.0 µg/mL; MFC = 1250.0 µg/mL	

The hydroethanolic extract (40:60, v/v) and EOs of *T. vulgare* and *T. balsamita* exhibited bacteriostatic effects on a broad range of Gram-positive bacteria, displaying MIC values ranging from 1 to 16 mg/mL for crude extracts, and 0.5–8 µg/mL for EOs, except for *L. monocytogenes* (17/1), which had a relatively higher MIC value (>64 mg/mL) (Table 4). Interestingly, the EO from *T. balsamita* showed promising bacteriostatic activity toward all the tested pathogenic Gram-negative bacteria, with *E. coli* (ATCC 25922) and *Y. enterocolitica* (O3 383/11) being the most prone to the EO (MIC value of 1 µg/mL) (Table 4). The authors suggested that the bacteriostatic activity of crude extracts and EOs could be due to the main volatile and phenolic compounds (camphor, α-thujone, and β-thujone), which mainly act by preventing the synthesis of nucleic acids, disrupting cytoplasmic membrane functions and deregulating bacterial metabolism (Bączek et al., 2017).

The aboveground methanolic extract of eight Serbian endemic medicinal plants, including *T. parthenium*, was tested against 16 pathogenic bacteria, such as *Escherichia coli*, *Staphylococcus aureus*, *S. pyogenes*, and *Pseudomonas aeruginosa*, among others, using the micro-well dilution method. Noteworthy, the methanolic extract from *T. parthenium* aerial parts displayed bactericidal activity against wound swabs-isolated bacteria, namely, *S. pyogenes* (MIC/MBC = 12.5/12.5 mg/mL) and *E. coli* (MIC/MBC = 25/50 mg/mL) (Stanković et al., 2016).

Rezazadeh et al. (2014) used the disc diffusion method to assess the antibacterial potency of *T. polycephalum* air-dried aerial parts EO against three Gram-positive and four Gram-negative bacteria. The essential oil was found to be active against the Gram-positive bacteria; *S. epidermidis* (ATCC 12228) (IZD = 28 mm), *Bacillus subtilis* (ATCC 6633) (IZD = 22 mm), and *S. aureus* subsp. *aureus* (ATCC 25923) (IZD = 25 mm). Likewise, three Gram-negative bacteria were also susceptible to the EO, namely, *E. coli* (ATCC 25922) (IZD = 19 mm), *Klebsiella pneumonia* (ATCC 10031) (IZD = 15 mm), and *Salmonella typhi* (PTCC 1609) (IZD = 15 mm), while *Shigella dysenteriae* (PTCC 1188) was relatively resistant (IZD = 5 mm) (Rezazadeh et al., 2014).

The α, β-unsaturated aldehydes (E)-2-decenal (**237**), (E)-2-undecenal (**238**), and (E,E)-2,4-decadienal (**239**) from the hexane extract of *T. balsamita* flowers, disclosed good antimicrobial activity against the uropathogenic Gram-negative bacteria *Proteus vulgaris* (MIC values of 12.5, 6.25, and 12.5 µg/mL, respectively). The α, β-unsaturated aldehydes also evidenced important activity toward five yeasts, namely, *saccharomyces cerevisiae*, *Candida utilis*, *Pityrosporum ovale*, *Penicillium chrysogenum*, and *trichophyton mentagrophytes* with MIC values within the range 1.56–25 µg/mL (Kubo and Kubo, 1995). The previous results justify the traditional uses of the genus *Tanacetum* for festering wounds, skin ulcers, and urinary tract infections. The promising MICs and MBCs suggested that the species in the genus warrant further studies to isolate its active components responsible for the bactericidal and fungicidal activities. Further investigations are also needed to screen the unexplored species for their antimicrobial activity.

A petroleum ether soluble fraction (PEE) from *T. vulgare* rhizome methanolic extract exerted dose-dependent toxic effects toward herpes simplex virus HSV-1 and HSV-2 (IC_50_ = 256.57 ± 9.27 and 126.29 ± 19.36 µg/mL, respectively) by disrupting the viral adsorption and uncoating. A bio-guided fractionation of PEE has led to the isolation of a spiroketal-enol ether derivative named (E)-2-(2,4-hexadiynyliden)-1,6-dioxaspiro [4.5]dec-3-ene using the thin layer chromatography (TLC) and ^1^H NMR. Intriguingly, the pure compound demonstrated virucidal activity on HSV-1 and HSV-2 (IC_50_ = 0.146 ± 0.013 and 0.127 ± 0.009 µg/mL, respectively) compared to the standard acyclovir (IC_50_ = 0.9 ± 0.01 and 0.7 ± 0.09 µg/mL, respectively). The significant activity of the compound was supposedly related to its capacity to alter viral gene expressions and therefore, the production of viral proteins such as envelope proteins (gG-2) (Álvarez et al., 2011; Álvarez et al., 2015).

The previous results partially validate the ethnomedicinal application of *Tanacetum* against sexually transmitted diseases, especially those caused by the herpes simplex virus (HSV). However, further investigations are needed to assess the antimicrobial potency of unexplored species on venereal conditions-causing microbes such as gonorrhea, syphilis, and trichomoniasis.

### 8.3 Anthelmintic activity

Since the 1940s, the overuse of synthetic drugs to boost productivity and control related livestock-infective helminths has led to parasitic resistances, in which pathogenic helminths have evolved elusive ways to circumvent the lethal effects of drug treatment (Dzoyem et al., 2020; McGaw and Abdalla, 2020; Doyle et al., 2022). Several *Tanacetum* taxa, including *T.vulgare*, *T. balsamita*, and *T. parthenium*, have traditionally been used as a vermifuge to control helminth infections in livestock, especially worms and tapeworms (Table 2).

The hydroethanolic extract and essential oil from *T. vulgare* aerial parts had significant *in vitro* schistosomicidal potency against *S. mansoni*. The crude extract causes 100% mortality of adult worms at doses of 100 and 200 µg/mL by decreasing motor activity and triggering tegumental damage, whereas the EO was only active at 200 µg/mL (Godinho et al., 2014).

A later study showed that the hydroalcoholic extract of *T. parthenium* aerial parts at a dose of 200 µg/mL killed all the adult parasites of *S. mansoni* after 48 h. The novelty of the study was the isolation and characterization of apigenin, santin, and parthenolide from the hydroalcoholic extract. Both flavones santin and apigenin were ineffective against *S. mansoni* adults up to 100 μM, whereas these sesquiterpene lactone parthenolide showed remarkable activity at 12.5 µM, causing 100% mortality, compared to the standard praziquantel (100% of mortality at 5 µM). The significant activity of parthenolide was purportedly related to its ability to reduce motor activity and induce tegumental alterations in schistosomes (de Almeida et al., 2016).

Moreover, the alcoholic extract of *T. vulgare* leaves and flowers reduced the viability of *Echinococcus granulosus* in a dose-and-time-dependent manner, causing 97.8% mortality after 30 min at 4 µg/mL (Omer, 2013). The previous findings validate the ethnomedicinal uses of *Tanacetum* species as a vermifuge, suggesting that *T. vulgare* could be a potential source for discovering safe and efficacious schistosomicidal compounds. However, further *in vivo* studies in *S. mansoni*-infected mice are required to validate the capacity of the plant to treat schistosomiasis.

### 8.4 Cytotoxic activity

The essential oil retrieved predominantly from the aerial parts of several species, including *T. balsamita* and *T. vulgare*, along with its main compounds, showed moderate cytotoxic properties, whereas minor compounds revealed remarkable *in vitro* cytotoxicity, indicating that the anticancer activity of the EOs may be driven by these constituents or a potential synergetic action of the entire mixture.

Gospodinova et al. (2014) used the MTT assay to examine the cytotoxic effects of the crude aqueous ethanolic extract from *T. vulgare* overground parts against the human breast cancer cell line (MCF7). The authors witnessed a time- and dose-dependent decrease in the cell viability with an IC_50_ value of 286.8 μg/mL after 72 h (Gospodinova et al., 2014). Moreover, five sesquiterpene lactones with the eudesmane skeleton from *T. vulgare* flowers dichloromethane extract were isolated and tested by Rosselli et al. (2012) for their *in vitro* cytotoxic activities against human lung cancer cells (A549) and hamster lung fibroblast cells (V79379A).Based on the ^13^C-NMR data, the authors identified these compounds as douglanin **(129)**, ludovicin A **(130)**, ludovicin B **(131)**, 1α-hydroxy-1-deoxoarglanine **(132)**, and 11,13-dehydrosantonin **(133)**. Accordingly, the isolated compounds disclosed significant time- and dose-dependent cytotoxic effects toward A549 with IC_50_ values ranging from 15.3 ± 0.1 to 59.4 ± 3.9 μM compared to the standard anticancer drug cisplatin 7.7 ± 2.1 μM. The cytotoxic properties of the five sesquiterpene lactones seem to be linked to their ability to induce apoptosis through the mitochondrial pathway. The authors concluded that these compounds could not disappointedly be used as anticancer drugs due to their non-selective nature against V79379A healthy cells (Rosselli et al., 2012). However, synthesizing derivatives of these compounds could be an effective approach to increase their selective distribution to cancer cells, while reducing their adverse effects on healthy normal cells.

Moreover, the essential oils obtained from the aerial parts of *T. vulgare* exhibited *in vitro* anticancer properties against both A-549 and healthy fibroblast cell line WS1 with IC_50_ values exceeding 200 μg/mL. Remarkably, colon adenocarcinoma cell lines DLD-1were the most susceptible to the EOs, with an IC_50_ value of 105 μg/mL. The authors indicated that the EOs’ preponderant compounds, namely, borneol, camphor, and 1,8-cineole had moderate cytotoxic potencies, while some minor volatile compounds such as β-pinene, caryophyllene oxide, β-caryophyllene, camphene, and γ-terpinene displayed promising activities (IC_50_ values ranging from 28 to 112 μg/mL) (Coté et al., 2017).

**FIGURE 7:**
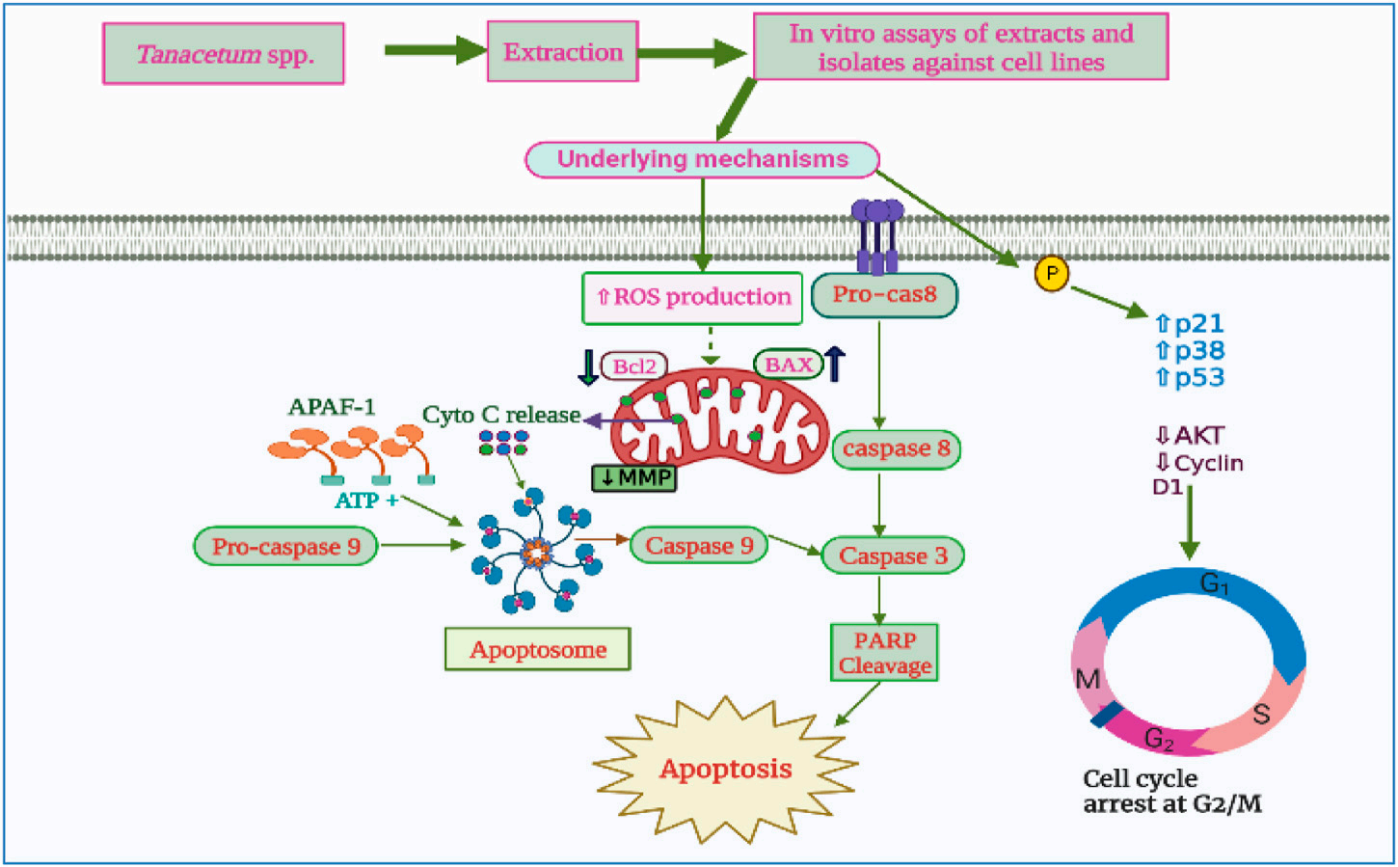
Anticancer activity of *Tanacetum* spp. extracts and isolates 2023^**©**^. The crude extracts and isolated compounds from *Tanacetum* triggered the intrinsic pathway of apoptosis in various cancer cells by increasing ROS production, Bax/Bcl2 ratio, cytochrome C release, and activating caspase cascade pathway. They also induced cell cycle block at the G2/M phase.

In another study, the cytotoxic effects of *T. vulgar*, *T. macrophyllum*, and *T. corymbosum* aerial parts crude chloroform extracts were investigated against human melanoma cells (A375), human cervical cancer cells (Hela), and Chinese hamster lung fibroblast cells (V79). The MTT test was performed to assess any decline in the cell viability of the tested cell lines. The authors recorded a dose-dependent reduction in cell viability, with HeLa cells being the most prone to the extracts displaying an inhibition rate ranging from 69.87% to 93.71% at the highest dosage of 200 μg/mL. The authors also reported the capacity of *T. vulgare* chloroform extract to induce apoptosis through the mitochondrial pathway, trigger DNA damage, and disrupt the cell cycle progression of V79 and A375 cells at the G2/M phase (Figure 7). In this study, the pronounced cytotoxic activity of *T. vulgare* was mainly associated with the presence of two trimethoxyflavone compounds, namely, eupatorin (41.92 μg/g dry weight of the plant) and eupatilin (0.31 μg/g dw plant) (Ivănescu et al., 2021).

On the other hand, nanoparticles (NP)-based drug delivery systems have proved several benefits, including accurate targeting of cancer cells, substantial drop in adverse effects and multi-drug resistance. In a recent *in vitro* study, Alipanah et al. (2021) stated that carvone (**160**) and EOs from *T. balsamita* exhibited weak cytotoxic effects toward breast cancer cells (MDA-MB468) and human melanoma (A375) cell lines with IC_50_ values of (3657.4, 6038.0 μg/mL) and (1312.1, 2323.6 μg/mL), respectively. Statistical analysis revealed that the cytotoxic activity of both carvone and EOs on A375 cells was not significantly different (*p* > 0.05), whereas EOs was slightly more efficient than carvone toward MDA-MB468 cells (*p* > 0.05). Subsequently, chitosan nanoparticles containing carvone and EOs of *T. balsamita* were prepared to improve their cytotoxic efficacies. Accordingly, chitosan nanoparticles containing *T. balsamita* EOs showed the best activity against both cell lines (A375 and MDA-MB468), with IC_50_ values of 85.3 and 240.1 µg/mL, respectively (Alipanah et al., 2021).

### 8.5 Antioxidant and hepatoprotective activities

The genus *Tanacetum* has been traditionally used to manage several oxidative stress-related diseases such as diabetes, hypercholesterolemia, and nerve system-related conditions. Various *in vitro* and *in vivo* studies have corroborated the ethnopharmacological uses of *Tanacetum* spp. as a traditional antioxidant remedy.

Pretreatment with the ethanolic extract from flowers and leaves of six Iranian *Tanacetum* taxa, namely, *T. tabrisianum*, *T. sonboli*, *T. chiliophyllum*, *T. hololeucum*, *T. kotschyi*, and *T. budjnurdense* at doses ranging from 10 to 100 µg/mL suppressed oxidative stress in hydrogen peroxide (H_2_O_2_)-treated K562 cells by increasing the intracellular glutathione (GSH), decreasing reactive oxygen species (ROS), glutathione peroxidase (GPx), and glutathione reductase (GR) activities (Esmaeili et al., 2010).

In another study, the pre-treatment and post-treatment of 70% methanolic extract of *T. parthenium* at doses of 80 and 120 mg/kg exhibited hepatoprotective effects in CCl_4_-induced liver damage in rats by substantially dropping LDL levels, total cholesterol, triglyceride, and glucose levels, compared to non-treated groups. The extract also increased HDL and albumin levels and brought antioxidant enzymes to near-normal ranges (AST, ALT, SOD, and GPx), indicating its capacity to prevent enzyme leakage and stabilize the cell membranes. The hepatoprotective effects were associated with tannins and flavonoids-rich methanolic extract (Mahmoodzadeh et al., 2017).

Elven guaianolides, germacranolides, and eudesmanolides sesquiterpene lactones from ethanolic extract of *T. parthenium* aerial parts, including parthenolide, 11,13dihydroparthenolide, 3-hydroxyparthenolide, santamarine, artemorin, and reynosin with α-methylene-γ-lactone moiety, were able to activate the nuclear factor E2-related factor 2 (Nrf2) through binding to antioxidant response element (ARE) in the genes’ promoter of mouse primary cortical neurons (Fischedick et al., 2012). Therefore, sesquiterpene lactones, tannins, and flavonoids from *T. parthenium* could be used as a template for developing new neurodegenerative and hepatoprotective drugs.

### 8.6 Antispasmodic activity

Ahmadnejad-Asl-Gavgani et al. (2022) investigated the anti-spasmodic properties of *T. balsamita* EO and its major component (carvone) on spasmogen-induced contractions in bovine ileum smooth muscle obtained from slaughtered bulls by adding nine cumulative concentrations from 0.10 to 1000 μg/mL to the tissue samples. Results showed that EO and its major constituent carvone remarkably reduced the *in vitro* spontaneous and spasmogen-induced contractions in ileum circular smooth muscle through inhibiting Ca^++^ channels in smooth muscle. The authors recommended *T. balsamita* as a strong candidate for treating hypermobility and intestinal spasms (Ahmadnejad-Asl-Gavgani et al., 2022).

### 8.7 Immunomodulatory activity

Polysaccharide-rich fractions from *T. vulgare* florets at doses 200–1600 μg/mL improved the immunomodulatory functions in murine J774.A1 macrophages by activating and increasing nitric oxide (NO) and reactive oxygen species (ROS) production, and tumor necrosis factor α (TNF-α).The polysaccharide fractions dose-dependently prevented erythrocyte hemolysis due to their ability to fix complement (serum proteins) compared to heparin, a complement fixing agent (Xie et al., 2007).

A flavonoid-rich extract from *T. parthenium* pollen grains at doses of 50 and 70 mg/kg significantly increased delayed-type hypersensitivity (DHR) and lymphocyte immune response in male Balb/C mice compared to non-treated animals (Jannesar et al., 2014). Previous studies suggest that Tansy polysaccharides can be used as a scaffold for new immunotherapeutic adjuvants.

### 8.8 Anti-inflammatory and antinociceptive activities

To bolster the ethnomedicinal claims of *T. balsamita* as a traditional anti-inflammatory remedy, Sharif et al. (2020) evaluated the *in vivo* acute anti-inflammatory activity of the aerial parts EOs in carrageenan-induced paw edema in a rat model at dosages of 100, 150, and 250 mg/kg. Mefenamic acid at 30 mg/kg served as the standard drug. Findings showed that the EO at 100 and 150 mg/kg failed to reduce carrageenan-induced paw edema compared to the reference drug. However, at a concentration of 250 mg/kg, the EO drastically (*p* < 0.05) lowers the carrageenan-induced rat paw edema production (54.91%), especially during the third hour of the test. The authors attributed the anti-inflammatory effects to the oxygenated monoterpene carvone (Sharif et al., 2020).

In a similar *in vitro* study, Karaca et al. (2009) stated that diethyl ether extract of *T. balsamita* above-ground parts at doses of 25, 50, and 100 mg/kg substantially suppressed carrageenan-induced paw edema formation in rats. The anti-inflammatory activity was linked to the presence of considerable amounts of flavonoids and their inhibitory action on inflammation mediators such as inducible nitric oxide synthase (iNOS) and cyclooxygenase-2 (COX-2) (Karaca et al., 2009).

Furthermore, a water extract containing 0.5% parthenolide from *T. parthenium* was investigated by Recinella et al. (2021) for its *in vitro* neuromodulatory and anti-inflammatory effects. The extract effectively reduced the release of prostaglandin (PGE2), extracellular dopamine, and IL-1β gene expression in hypothalamic Hypo-E22 cells, while increased dopamine transporter (DAT), IL-1β, IL-10 and brain-derived neurotrophic factor (BDNF) genes expression. The authors concluded that targeting dopaminergic pathways could be an effective therapeutic approach in preventing and managing migraine attacks (Recinella et al., 2021).

Moreover, extracts of *T. parthenium* (ethanol, acetone and a mixture of water, acetone, and alcohol) depleted of the sesquiterpene lactone parthenolide (a skin sensitizer) inhibited pro-inflammatory enzymes such as phosphodiesterase-3 and 4 and 5-lipoxygenase in murine macrophages. Extracts also showed the ability to inhibit pro-inflammatory mediators, including TNF-α, nitric oxide, PGE2, IFN-γ, IL-2, and IL-4. According to the authors, extracts depleted of parthenolide were effective in alleviating inflammation without stimulating the immune system (Sur et al., 2009).

On the other hand, several straightforward assays, including the writhing test, tail-flick test, formalin test, and hot-plate test, have been used to validate the antinociceptive properties of *T. balsamita* as a traditional painkiller remedy. Sharif et al. (2020) performed the hot plate test to assess the *in vivo* anti-nociceptive activity of *T. balsamita* aerial parts EOs at the dosages of 50, 100, 200, and 400 mg/kg. The authors stated that the EOs at 25 and 50 mg/kg did not affect the reaction time to the heat source compared to the conventional drug morphine. However, at a dose of 100 mg/kg, the EO displayed significant anti-nociceptive activity by delaying the response time to the thermal stimulant. The authors reported that the antinociceptive mechanism of the essential oil at this concentration was most likely non-opioid (Sharif et al., 2020).

### 8.9 Insecticidal, larvicidal, and repellent activities

Several studies have validated the traditional uses of *Tanacetum* spp. as insecticidal, larvicidal, and repellent agents. Attighi Lorestani et al. (2013) investigated the fumigant toxicity of *T. balsamita* essential oil against eggs and adults of *Callosobruchus maculatus* F. (Cowpea weevil) at doses ranging from 5.3 to 17.4 μL/Lair for eggs, and 5.12–20.24 μL/Lair for adults. The authors found that the essential oil had dose- and time-dependent fumigant toxicity, with the treated adults being generally more vulnerable than eggs. The lowest concentration of 5.12 μL/lair caused a mortality rate of 59.82% in adults, while a mortality rate of 22.21% was recorded in eggs at the lowest concentration of 5.3 μL/Lair after 72 h of exposure. The authors attributed the significant fumigant toxicity to the major constituents of *T. balsamita*, such as camphor, bornyl acetate, pinocarvone, and terpinolene (Attighi Lorestani et al., 2013).

Similarly, Gokturk et al. (2017) revealed that *T. balsamita* EO at the dosage of 20 μL/Petri causes a mortality rate of 34.4% in *Hyphantria cunea* (Drury) (White Butterfly) after 96 h of exposure (Gokturk et al., 2017). In another study, Grulova et_al., 2017 tested the repellency effects of the EOs of six medical plants, including *T. balsamita* toward *Rhopalosiphum padi* L, a major pest in cereal crops, using doses ranging from 0.1% to 1%. Costmary EO disclosed a dose-dependent repellency effect, which was more pronounced at the highest concentration of 1.0% (Grulova et al., 2017).

Moreover, CO_2_ essential oil and extract of *T. parthenium* aerial parts exhibited antifeedant and growth inhibition on *Spodoptera littoralis* (Boisduval) larvae with LD_50_ values of 0.05 and 0.11 µL/g, respectively (Pavela et al., 2010). Additionally, the 80% ethanolic extract of powdered *T. parthenium* was tested by Erdoğan and Yıldırım (2016) on the green peach aphid *Myzus persicae* Sulzer using leaf-dipping and spraying methods. The extract showed aphidicidal activity against nymphs and adults when diluted at 6% and 12%. Mortality rates of 82% and 88% were observed against nymphs, and 75% and 88% against adults, respectively. In the spraying method, the extract at 6% and 12% caused 70% and 87% of mortality among adults of *M. persicae* Sulzer (Erdoğan and Yıldırım, 2016).

### 8.10 Anticholinesterase activity

The acetonitrile extracts of leaf and flowers of several *Tanacetum* species were investigated for cholinesterase inhibitory activity at 100 µg/mL. The extracts significantly inhibited acetylcholinesterase (AChE) with *T. argenteum* subsp. *flabellifolium* having the highest inhibition (96.68% ± 0.35%), whereas a moderate activity was observed against butyrylcholinesterase (BChE) (Orhan et al., 2015).

## 9 Clinical evidence

According to the Cochrane library database, there are 36 documents dealing with clinical studies of the genus *Tanacetum*, especially *T. parthenium* (feverfew).The first report about the prophylactic properties of feverfew surfaced in British Health Magazine in 1978, documenting the case of a patient suffering from migraine attacks since the age of 16. At the age of 68, she commenced taking three feverfew leaves daily for 10 months, and her terrible headache entirely ceased (Pareek et al., 2011). Afterward, several double-blind, randomized controlled trials (RCTs) have been enrolled to examine the safety and the clinical effectiveness of feverfew-based nutraceutical formulations for episodic migraines without aura (as a symptomatic treatment) and as a prophylactic therapy for migraine with aura. For instance, a double-blind, placebo-controlled clinical study (n = 57) was undertaken by Palevitch et al. (1997) to explore the efficacy of feverfew leaves as a prophylactic measure towards migraine attacks and their commonly associated symptoms such as nausea, vomiting, and light sensitivity. Results revealed that feverfew substantially reduced the severity of pain by 4.27 scale points compared with the placebo. Additionally, the authors reported a noticeable decrease in the intensity of the typical migraine symptoms, including nausea, vomiting, and sensitivity to light and sound (Palevitch et al., 1997). However, some patients claimed incapacitating headaches as a result of the quick withdrawal of feverfew after switching to the placebo medication.

In a recent study, oral administration of a fixed dose of Partena (2 tablets per day), consisting of riboflavin, magnesium, CQ10, and *T. parthenium*, significantly dropped headache frequencies (50%) among pediatric patients having tension-type headaches (TTH) (n = 91) after 16 weeks. However, 4.4% of the patients claimed to have gastrointestinal symptoms and interrupted the treatment (Moscano et al., 2019).

A double-blind, placebo-controlled, multicenter open-label randomized controlled trial (RCT) was conducted by Diener et al. (2005) to assess the prophylactic effects of feverfew CO_2_-extract in patients (n = 218) diagnosed with migraine with or without aura according to the IHS criteria. Data from 170 patients showed that extract at a dose of 6.25 mg significantly decreased the monthly migraine frequency attacks from 4.8 to 2.9 (*p* = 0.0456) compared to a placebo (4.8–3.5) between weeks 5 and 12 (Diener et al., 2005).

Evidence from studies showed that the anti-migraine properties of feverfew are likely associated with the stimulation of cytokines, and the suppression of nitric oxide production, serotonin release from platelets, nuclear factor-kappa B(NF-κB), and CGRP (calcitonin gene-related peptide) from the trigeminovascular system (Moscano et al., 2019).

However, we noticed that the studies were relatively small in size (ranging from 17 to 218 participants). Therefore, their statistical analyses could be biased, due to the hazards of random chance, which can make small sample sizes prone to overestimation. Therefore, long-term clinical trials with relatively larger sizes and rigorous methodologies are required to validate the efficacy and the safety of feverfew in preventing and treating migraine attacks.

## 10 Toxicity

*Tanacetum* spp. extracts have been evaluated by several research groups for acute or/and chronic toxicity and safety. Yousefzadi et al. (2009) evaluated the cytotoxic effect of *T. balsamita* aerial parts EOs towards monkey kidney (Vero) and human fetal skin fibroblast (HFSF) cell lines using the MTT assay. Accordingly, a weak cytotoxic effect has been noticed against both cell lines with IC_50_ values of 2500 and 1250 μg/mL, respectively. The results from this study may indicate the safe use of the plant’s EOs. However, further *in vivo* acute and chronic toxicity studies are required to validate the plant’ safety (Yousefzadi et al., 2009).

Lahlou et al. (2008) stated that oral and intraperitoneal administration of single doses (0–13 g/kg and 0–4.5 g/kg, respectively) of an aqueous extract from *T. vulgare* leaves for 90 days had insignificant acute and chronic toxicity in rodents due to relatively no-observed adverse effect levels (NOAEL) values (9.0 g/kg and 1.5 g/kg, respectively) and absence of noticeable effects on rats’ hematological and biological parameters after 90 days in rats (Lahlou et al., 2008). The potential acute and chronic toxicities of extracts and isolated compounds, especially sesquiterpene lactones, should be further investigated.

## 11 Potential use of the genus *Tanacetum* as natural food preservative

The widespread distrust towards synthetic additives puts increasing pressure to seek natural and health-beneficial substitutes for chemical additives (Karimi et al., 2021). The use of plant-based extracts and essential oils as natural antimicrobial and antioxidants has been corroborated by a plethora of studies, which constitute a renewable supply of active agents for eco-friendly food packaging (Carocho et al., 2014; Karimi et al., 2021).

In this sense, Khodayari et al. (2019) investigated the effect of the poly (lactic acid) composite film containing 2% *T. balsamita* EO (TBE), 1% cellulose nanocrystals composite (CNC), and 2% propolis ethanolic extract (PEE) on vacuum-packed sausages shelf life. Results revealed that the prepared film disclosed potent antimicrobial capacity and significantly prolonged cooked sausages’ shelf life by 50 days of refrigerated storage. They also showed that the active film was especially active against the Gram-positive bacteria, with *B. cereus* being the most susceptible. In the same study, the authors witnessed a synergic effect between *T. balsamita* EO (TBE) and propolis ethanolic extract and reported that TBE operated as a plasticizer on the blend (Khodayari et al., 2019). In another study, Nobakht and Moghaddam, (2013) reported the capacity of 1.5% and 2% of costmary added to laying hens’ diets to improve their overall performance, blood biochemical parameters, and egg characteristics (Nobakht and Moghaddam, 2013).

Altogether, the genus *Tanacetum* might be a valuable repository of chemical compounds that could be exploited as natural food additives and as a platform for biodegradable active packaging development in the food industries.

## 12 General discussion

**FIGURE 8:**
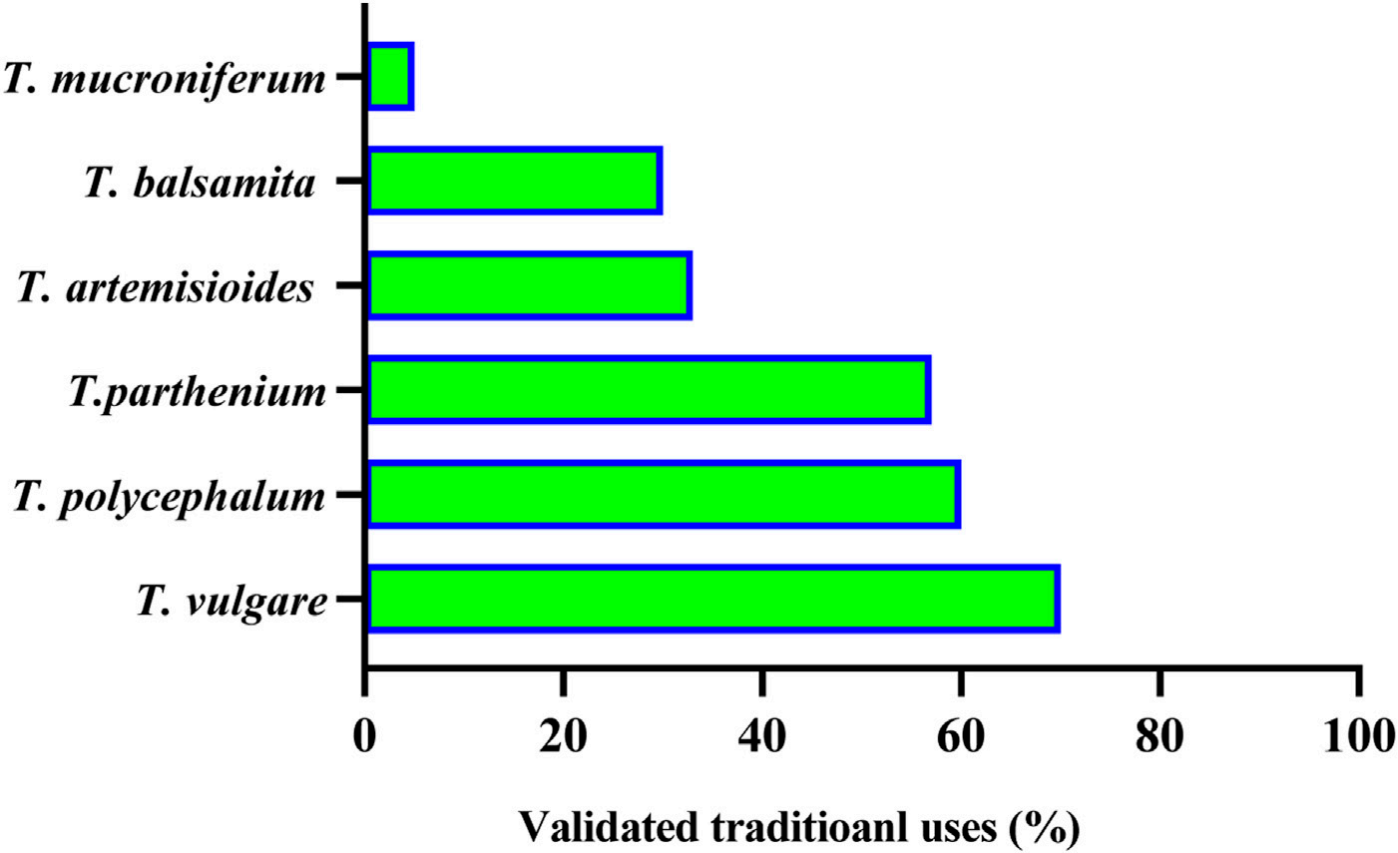
Validated ethnomedicinal uses per *Tanacetum* species (%).

*Tanacetum* species carry a long history of traditional uses in various fields, including medicine, cosmetics, agriculture, and cuisines. Overall, various ethnomedicinal applications have been recently supported through *in vitro* and *in vivo* pharmacological studies. For instance, the use of the several *Tanacetum* species, including *T. vulgare*, *T. balsamita*, and *T. parthenium* for festering wounds, skin ulcers, urinary tract infections, and gastrointestinal and venereal conditions is evident from their antibacterial, antiviral, and antispasmodic activities. The use of *T. vulgare* for oral hygiene has been validated by the *in vitro* inhibition of cariogenic oral bacteria, mainly *Streptococcus mutans*. The use of *T. vulgare* and *T. parthenium* as a vermifuge has been confirmed by *in vitro* anthelmintic studies. In addition, the use of *T. vulgare*, *T. balsamita*, and *T. parthenium* against inflammation, pain, and fever was backed up by their anti-inflammatory and antinociceptive activities, mainly through inhibiting pro-inflammatory mediators’ release, such as nitric oxide, TNF-α, PGE_**2**_, IL-2, IL-4, and IFN-γ. Ethnopharmacological studies have also documented the use of several *Tanacetum* species for liver disorders. Pharmacological investigations have confirmed this usage by establishing its hepatorestorative action against hepatotoxicity induced by various substances, such as CCl_4_, in animal studies. The use of *T. vulgar*, *T. balsamita*, *T. macrophyllum*, and *T. corymbosum* for cancer treatment has been supported by cytotoxic studies against various cancer cell lines. The ethnomedicinal use of *Tanacetum* spp. for diabetes management has been proven by its α-glucosidase, α-amylase, and protein tyrosine phosphatase-1B (PTP-1B) activity, as well as in a STZ-induced Sprague-Dawley rat’s model. However, the use of the *Tanacetum* spp. for bile acid deficiency, arthritis, gout, rheumatism, anemia, and as a litholytic, diaphoretic, and antivenom has not yet been checked. Table 5 and Figure 8 include further details about the validated traditional uses to establish a basis for future studies and help to fulfill the research gaps. The following paragraphs provide additional insights into the validated traditional uses and research gaps.

**TABLE 5:** Validated traditional uses per *Tanacetum* species.

Species	Traditional uses	Modern studies			References
		Validated	*In vitro*	*In vivo*	
*T. balsamita*	Diabetes	**✓**	**✓**	**✕**	Vukic et al. (2022)
	Antispasmodic	**✓**	**✓**	**✕**	Ahmadnejad-Asl-Gavgani et al. (2022)
	Antipyretic	**✕**	**✕**	**✕**	Hussain et al. (2018)
	Hepatoprotective	**✓**	**✕**	**✓**	Rusu et al. (2005)
	Wound healing	**✕**	**✕**	**✕**	Sõukand and Pieroni (2016)
	Anti-inflammatory	**✓**	**✓**	**✓**	(Sharif et al., 2020; Servi et al., 2021)
	Antiallergic	**✕**	**✕**	**✕**	Hassanpouraghdam (2009)
	Migraine	**✕**	**✕**	**✕**	Hassanpouraghdam et al. (2022)
	Bile acid deficiency	**✕**	**✕**	**✕**	Ghirardini et al. (2007)
	Anticancer	**✓**	**✓**	**✕**	Alipanah et al. (2021)
	Arthritis	**✕**	**✕**	**✕**	Juan-Badaturuge et al. (2009)
	Anthelmintic	**✕**	**✕**	**✕**	Agelet et al. (2000)
	Antihypertensive	**✕**	**✕**	**✕**	Bouhlal et al. (2017)
	Cholecystitis	**✕**	**✕**	**✕**	Guarino (2008)
*T. vulgare*	Anthelmintic	**✓**	**✓**	**✕**	(Omer, 2013; Godinho et al., 2014)
	Rheumatism	**✕**	**✕**	**✕**	Tribess et al. (2015)
	Fever	**✕**	**✕**	**✕**	Petrov (2016)
	Epilepsy	**✕**	**✕**	**✕**	Petrov (2016)
	Type 1 diabetes	**✓**	**✕**	**✓**	Mohseni-Salehi-Monfared et al. (2010), Chaachouay et al. (2019a)
	Anemia	**✕**	**✕**	**✕**	Chaachouay et al. (2019b)
	Hypercholesterolemia	**✓**	**✕**	**✓**	Azonov et al. (2008), Chaachouay et al. (2019a)
	Anti-inflammatory	✓	✓	✓	Schinella et al. (1998), Holetz et al. (2002), Coté et al. (2017)
	Neurological conditions	✓	✓	✓	Daneshmand et al. (2016), Ak et al. (2021)
	Migraine	**✓**	**✕**	**✓**	Schinella et al. (1998)
	Venereal conditions	**✓**	**✓**	**✕**	Álvarez et al. (2015)
	Kidney diseases	**✕**	**✕**	**✕**	Shikov et al. (2014)
*T. parthenium*	Gastric disorders	**✓**	**✓**	**✕**	Gholami et al. (2014); Tribess et al. (2015)
	Fever	**✓**	**✓**	**✓**	Lans et al. (2006); Sur et al. (2009)
	Toothache	✓	✓	✓	Jain and Kulkarni (1999), Delfan et al. (2014)
	Litholytic (Urolithiasis)	**✕**	**✕**	**✕**	Ahmed et al. (2016)
	Rheumatism and arthritis	**✕**	**✕**	**✕**	Vokou et al. (1993)
	Sedative	**✓**	**✓**	**✓**	Vokou et al. (1993), Mannelli et al. (2015)
	Diaphoretic	**✕**	**✕**	**✕**	Vokou et al. (1993)
*T. artemisioides*	Diabetes	**✕**	**✕**	**✕**	Ullah et al. (2019)
	High blood pressure	**✕**	**✕**	**✕**	Ullah et al. (2019)
	Migraine pain and fever	**✓**	**✕**	**✓**	Bukhari et al. (2007), Ibrar and Hussain (2010)
	Ringworm	**✕**	**✕**	**✕**	Hussain et al. (2010), Hussain et al. (2018)
	Hepatitis	**✕**	**✕**	**✕**	Ibrar and Hussain (2010)
	Headache	**✓**	**✓**	**✕**	Ahmad et al. (2004)
*T. polycephalum*	Gastroenteritis	**✕**	**✕**	**✕**	Mosaddegh et al. (2012)
	Hemorrhoids and inflammation	✓	✓	**✕**	Coté et al. (2017)
	Analgesic	**✓**	**✕**	**✓**	Azizi et al. (2020)
	Cancer	**✓**	**✓**	**✕**	Karimian et al. (2015)
	Cold and flu	**✕**	**✕**	**✕**	Kawarty et al. (2020)
*T. mucroniferum*	Inflammation	**✕**	**✕**	**✕**	Türker (2018)
	kidney problems	**✕**	**✕**	**✕**	Türker (2018)
	Influenza	**✕**	**✕**	**✕**	Türker (2018)
	Typhoid fever	**✕**	**✕**	**✕**	Van Wyk and Gorelik (2017)
	Antivenom for scorpion	**✕**	**✕**	**✕**	Van Wyk and Gorelik (2017)
	Infantile spasms	**✕**	**✕**	**✕**	Van Wyk and Gorelik (2017)

First of all, most of the reported antimicrobial studies focused on crude extracts rather than isolated compounds. Therefore, it is likely that the reported antimicrobial activity is due to a synergistic effect of the active metabolites present in the plant extracts. Accordingly, species in the genus warrant further investigations to isolate potentially active compounds that could be involved in the bactericidal, virucidal and fungicidal properties. Moreover, most of these studies have tended to use disc diffusion assay, which is unreliable for measuring antimicrobial activity since the compounds’ polarity impact how effectively they diffuse into the polar agar medium and consequently alter the inhibition zone size. In contrast, agar dilution and broth microdilution methods enable precise quantitative conclusions by determining MIC values for antimicrobials (Khatib et al., 2022b). Therefore, they are highly recommended in future studies for regular antimicrobial susceptibility testing. In line with the traditional uses of the genus *Tanacetum* against venereal conditions, the anti-infective activities of crude extracts and isolated compounds may potentially consider microbial threatening diseases, including the resistant strains of gonorrhea *Neisseria gonorrhoeae and* bacteria causing sexually transmitted infections (STI) such as *Chlamydia Trachomatis* and *Mycoplasma genitalium*, and so forth.

Several taxa from the genus, especially *T. balsamita* and *T. vulgare*, are still appreciated in the traditional cuisine of several countries, including Italy and Russia, owing to their pleasant aroma and bitter taste. Perhaps their usage could also be justified by their richness in minerals, vitamins (A, D, E, K), mono- and polysaccharides (rhamnose, galactose, glucose, mannose, apiose, and xylose), and other crucial elements of a balanced diet. Previous studies highlighted the capacity of these metabolites to protect many target tissues against oxidative stress-induced diseases (e.g., neurological, cardiovascular, and liver diseases, among others) (Uberti et al., 2014; Zeng et al., 2017). Hence, ROS and free radicals scavenging, ferric reducing capacity, as well as the rise in physiological antioxidants, including SOD, AST, ALT, HDL, and GPx, could be attributed to the antioxidant vitamins and polyphenolic content.

Ethnopharmacological studies reported that *T. artemisioides* whole plant is used indigenously for high blood pressure and neurological conditions. Though scientific reports on *T. artemisioides*’ neuroprotective potency are still scarce or even missing, Ahmad et al. (2004) reported the isolation of two newly identified ceramides called tanacetamide A and B from a methanolic extract of *T. artemisioides*. The newly identified compounds exhibited remarkable *in vitro* acetylcholinesterase inhibitory properties, with IC_50_ values of 67.1 ± 1.5 and 74.1 ± 5.0 μM, respectively, compared to the standard galanthamine (IC_50_ = 8.5 ± 0.0001 μM) (Ahmad et al., 2004).

Indeed, several hypotheses have been put forward to explain the pathogenesis of Alzheimer’s disease. One of them is known as the Cholinergic Hypothesis, describing the inhibition of Cholinesterase (ChE) enzyme family (Kumar et al., 2022). Thus, these metabolites may lead to a breakthrough in disease treatment. However, further studies are required to evaluate the potency of these ceramides to interfere with the amyloid-β (Aβ) pathway. In addition, drug-drug interactions, particularly those involving anticoagulants and antiplatelet drugs, should be carefully examined, as well as the risk-to-benefit ratio for isolated compounds can be established through long-term multicenter trials with large sample sizes and rigorous methodologies. Besides, the structure-activity relationship tool (SAR) can be used to modify and optimize these compounds to compete with current market drugs.

Hypertension is a global health problem involving the interaction of genetic and environmental factors. Indeed, it is associated with an increased risk of stroke, cardiovascular and kidney diseases (Donfack et al., 2021). Ethnobotanical studies highlighted the extensive usage of *T. artemisioides* for high blood pressure. Although the available data revealed a critical shortage on *Tanacetum’* antihypertensive activity, a study showed that the sphingosine-type tanacetamide isolated from an aqueous extract of *Vitex cienkowskii* stem bark displayed potent vasorelaxant activity through increasing the endothelial production of nitric oxide (NO) and the activation of vascular smooth muscle soluble guanylate cyclase (sGC) (Dongmo et al., 2011). Therefore, the ethnomedicinal usage of *T. artemisioides* as a hypotensive agent could be attributed to the presence of tanacetamide (A-D). For this reason, *in vivo* and *in vitro* studies on the antihypertensive potency of *T. artemisioides* crude extracts and isolated tanacetamide (A-D) are of utmost necessity, especially against the angiotensin-converting enzyme (ACE).

*T. vulgar*, *T. macrophyllum*, and *T. corymbosum* extracts and isolated compounds displayed promising *in vitro* cytotoxic activity against various cancer cell lines through triggering the intrinsic pathway of apoptosis, increasing ROS production, Bax/Bcl2 ratio, cytochrome C release, and activating caspase cascade pathway. They also showed the capacity to induce cell cycle block at the G2/M phase. Sesquiterpene lactones with the eudesmane skeleton, including douglanin **(129)**, ludovicin A **(130)**, ludovicin B **(131)**, 1α-hydroxy-1-deoxoarglanine **(132)** have been correlated with the cytotoxic activity of *Tanacetum* spp. suggesting, therefore, that these metabolites could be behind the significant cytotoxic activity of these species, as well as solid leads of anticancer compounds.

A prominent hepatoprotective activity of *Tanacetum* spp. has been noticed by restoring antioxidant enzymes, biochemical factors, lipid peroxidation, and liver enzymes in animal models. In fact, a careful examination showed that high doses have been used in the reported studies (400 mg/kg). When used in such doses, it may cause harmful or severe adverse effects on humans. Thus, further studies with tolerable doses to be used in human subjects are required. The hepatoprotective tests may also consider noting the dose and range utilized the animal type, number, sex, the drug vehicle, and the method of anesthesia and/or killing (appropriate).

In conclusion, *Tanacetum* spp. and their isolated compounds showed broad and significant therapeutic merits both *in vitro* and *in vivo*. However, numerous studies had some gaps to be addressed by more studies. The current review help-build a foundation for further research.

## 13 Conclusion and recommendations

The genus *Tanacetum* has been ethnopharmacologically used to treat numerous diseases such as arthritis and fever, hypertension, nausea, kidney problems, dyspepsia, stomach pain and bloating, diabetes, festering wounds, flu and cold, and migraine. Several pharmacological studies have supported enormous traditional uses such as anthelmintic, antidiabetic, anticancer, antioxidant, insecticide, and hepatoprotective activities as well as against skin ulcers, festering wounds, urinary tract infections, and sexually transmitted diseases. An extensive literature search using various online search engines showed that ethnobotanical data for only 16 taxa **(10%)** out of 160 accepted were available. Hence, further ethnobotanical surveys should be undertaken to document and preserve the folkloric knowledge of the remaining species.

Moreover, several species are reportedly under critical threat of extinction by the International Union for Conservation of Nature (IUCN), especially *T. ptarmiciflorum*, *T. oxystegium*, and *T. oshanahanii*, and were included on the critically endangered species red list. Also, there were only eight species **(5%)** out of 160 accepted taxa from the genus evaluated by the International Union for Conservation of Nature (IUCN) for their statuses. Therefore, a large-scale risk assessment and *ex-situ* and *in-situ* measures are necessary to ensure the sustainability of the genus and prevent its extinction.

Ceramides such as tanacetamide A-D (**72-75**), pyrethrins I and II (**113, 114**), cinerin I and II (**115, 116**), and jasmolin I and II (**117, 118**)) could serve as chemotaxonomic markers of the genus *Tanacetum* due to their restricted occurrence within the genus *Tanacetum*. They may serve along with DNA barcoding methods as crucial tools to resolve the controversial infrageneric classification of the genus *Tanacetum* and ensure its quality control. Despite their relatively toxic nature, these compounds exhibit numerous interesting pharmacological properties such as anti-acetylcholinesterase, antihypertensive, antimicrobial, neuroprotective and cytotoxic activities. Thus, further investigations should be done to investigate the unexplored biological activities of these compounds in line with traditional uses of *Tanacetum* species.

The anticancer activity of crude extracts and isolated metabolites, especially sesquiterpene lactones, are based on preliminary cytotoxic studies. While providing valuable insight into the cellular mechanisms underlying the anticancer effects, as well as initial data on their toxicity and selectivity, they do not fully capture the complexity of the *in vivo* environment, especially the intercellular and tissue interactions, which can influence drug metabolism, distribution, and toxicity. Thus, *in vivo* studies are needed to validate the *in vitro* studies and determine the pharmacological relevance of these metabolites, while providing insight into their efficacy, safety, and pharmacokinetics, as well as their potential impact on the host organism. Similarly, the antidiabetic studies are mostly based on *in vitro* models. Therefore, further *in vivo* studies on animal models are needed to identify potential therapeutic targets and evaluate the effectiveness and safety of extracts and isolated metabolites from the genus species.

Finally, some studies, especially those evaluating the anti-inflammatory, antimicrobial, and insecticidal activities of *Tanacetum* spp. are poorly reported due to the lack of positive control and high doses used, which make their findings less reliable.

## Data Availability

The original contributions presented in the study are included in the article/Supplementary Material, further inquiries can be directed to the corresponding authors.

## Abbreviations

Ace, Acetone; ALT, Alanine transaminase; AmOx, Ammonium oxalate; AST, Aspartate aminotransferase; Bax, Bcl-2 Associated X-protein; Bcl2, B-cell lymphoma 2; CC, Column chromatography; CH2Cl2, Dichloromethane; DPPH, 1,1-diphenyl-2-picrylhydrazyl; Dw, Dry weight; EO, Essential oil; EtOAc, Ethyl acetate; FRAP, Ferric ion reducing antioxidant power; HDL, High-density lipoprotein; HPLC, High-Performance Liquid Chromatography; IHS, The official International Headache Society; i-PrOH, Isopropanol; IUCN, The International Union for Conservation of Nature; IZD, Inhibition zone diameter; LDL, Low-density lipoprotein; MCP-1, Chemoattractant protein-1; MeOH, Methanol; MFC, Minimum fungicidal concentration; NF-κB, Nuclear factor kappa B; PEE, Petroleum ether; SFE, Supercritical fluid extraction; SOD, Superoxide dismutase; TGF-β1, Growth factor beta; TLC, Thin Layer Chromatography.
